# A meta‐analysis of forgiveness education interventions’ effects on forgiveness and anger in children and adolescents

**DOI:** 10.1111/cdev.13771

**Published:** 2022-04-22

**Authors:** Hannah Rapp, Jiahe Wang Xu, Robert D. Enright

**Affiliations:** ^1^ 5228 Department of Educational Psychology University of Wisconsin‐Madison Madison Wisconsin USA; ^2^ International Forgiveness Institute, Inc Madison Wisconsin USA

## Abstract

Forgiveness education interventions instruct children and adolescents in understanding forgiveness and its role in healthy relationships. In this meta‐analytic review, 20 studies involving 1472 youth (51% female; *M*_age_ = 11.66) from 10 countries (studies: 40% North American, 25% East Asian, 20% Middle Eastern, 15% European) were retrieved to determine forgiveness education interventions’ effects on youth outcomes. Hedges’ *g* and confidence intervals (CIs) were used to assess treatment effects. Findings suggest that forgiveness education interventions have a significant positive effect on forgiveness (*g* = 0.54, 95% CI [0.36, 0.73]) and anger (*g* = 0.29, 95% CI [0.11, 0.47]). Results lend support to the idea that children and adolescents who experience hurt from the unjust actions of others may benefit from learning about the process of forgiveness.

**Abbreviations**
CI: confidence interval
ES: Effect size
FE: forgiveness education
ICC: intraclass correlation coefficient
PBIS: Positive Behavioral Interventions and Support
RE: Random effects

There are different approaches to understanding forgiveness. Forgiveness is a prosocial motivational change that follows an offense (McCullough, 2001), however, what that prosocial change entails varies by forgiveness scholar. In one common forgiveness approach, prosocial motivations manifest as decreases in avoidance of and revenge toward the person who offended and increases in the desire and actions to rebuild a relationship with them (McCullough et al., 1998). In another common forgiveness approach, a person, who is unjustly hurt by another, abandons resentment toward the offender, and instead offers goodness and compassion, even if the offender does not deserve these gifts (Enright & Fitzgibbons, 2015, p. 32; North, 1987). In this approach, offering goodness and compassion to the person who offended may not be exemplified by rebuilding the relationship or even interacting with the person who offended (sometimes for one's own safety). Rather, a person can abandon resentment and offer goodness in a variety of other ways, such as thinking of those who offend as possessing worth, acting considerately toward them, feeling favorable toward them, or hoping they succeed in life, and similar activities.

Furthermore, some forgiveness researchers support that people have two different processes or types of forgiveness: decisional forgiveness (making behavioral intention statements) and emotional forgiveness (replacing negative emotions with positive feelings (Worthington, 2005, p. 11). In contrast, other forgiveness researchers maintain that there is one more holistic forgiveness process that includes decisions and emotions, as well as behaviors, as part of the moral virtue of forgiveness (Enright & Fitzgibbons, 2015). Researchers generally agree that forgiveness is not the same as reconciling or restoring a broken relationship because forgiveness is not contingent on a continued relationship with the person who committed the offense (Enright & Fitzgibbons, 2015, p. 44; Hui & Chau, 2009). Thus, forgiveness does not necessarily include any interaction or contact with the offender. In addition, forgiveness is not the same as forgetting, condoning, or excusing an offense (Exline et al., 2003; Worthington, 2005, p. 11).

## Effects of forgiveness and anger on children and adolescents

As with adults, children and adolescents experience interpersonal conflicts. A child or adolescent may respond to an offense by retaliating. However, revenge as a conflict resolution strategy is harmful to relationships and can make it difficult for a child to maintain strong friendships (Rose & Asher, 1999). In comparison, forgiveness is important to the interpersonal relationships and the psychological well‐being of children and adolescents (Flanagan et al., 2012; van der Wal et al., 2017). Children who forgive are more accepted by their peers (van der Wal et al., 2017). Additionally, forgiveness has been shown to reduce anger among children and adolescents (Taysi et al., 2015; Watson et al., 2017).

Experimental studies of forgiveness interventions for children and adolescents show that anger is particularly connected to forgiveness (Enright et al., 2007; Holter et al., 2008; Taysi & Vural, 2015). In addition, two meta‐analytic reviews with the majority of adult study samples, which explore the correlates of interpersonal forgiveness (Fehr et al., 2010; Riek & Mania, 2012), report medium to strong correlations between forgiveness and anger. Thus, it is also important to look at the effects of anger on children and adolescents. Persistent anger can lead to antisocial behavior such as increased violence and aggression (Aseltine et al., 2000; Hawes et al., 2016). Anger can also lead to academic difficulty (Loveland et al., 2007; Strauss et al., 1987; Wiesner & Windle, 2004). In a longitudinal study following children at the ages of 6 and 9, Zhou et al. (2010) found that anger was associated with externalizing behaviors that predicted lower average grades. Among children and adolescents, persistent anger is also associated with depressive symptoms (Carey et al., 1991; Taysi & Vural, 2015). Additionally, anger can lead to a behavior called co‐rumination, which is especially prevalent among adolescent girls (Rose, 2002; Rose et al., 2014). Co‐rumination is defined as excessively re‐opening or discussing past problems. A study by Guarneri‐White et al. (2015) revealed that co‐ruminating with a same‐sex best friend moderates the relationship between being victimized by a peer and depressive symptoms. An additional study explains that when parents engage in co‐rumination with their adolescent child, they can cause their child to exhibit symptoms of anxiety and depression (Waller & Rose, 2013).

## Forgiveness education interventions

In recent years, strong evidence for the effectiveness of forgiveness interventions among adults, combined with the scholastic desire for social‐emotional learning curriculums, has led to the implementation of forgiveness education interventions in schools or group settings. The first published calls for forgiveness education are in Enright (2001) and in Enright et al. (2003). The present study defines a forgiveness education intervention as a group intervention that teaches children and adolescents about what forgiveness is and the benefits of forgiveness (i.e., its role in healthy relationships) as well as the process(es) of forgiveness. All forgiveness education interventions were created for children and adolescents (elementary through high school) to be administered in classrooms.

What are the differences between therapeutic forgiveness interventions and forgiveness education interventions? To start, therapeutic forgiveness interventions are not designed for schools although they can take place in group settings. In addition, forgiveness education interventions have the central goal of helping youth to develop the knowledge and skill of forgiveness to become more socially and mentally healthy people. In this way, forgiveness education interventions are more like social‐emotional learning programs than they are like therapeutic interventions, although they may have therapeutic components. Social‐emotional learning (SEL), refers to the development of specific skills and competencies that students need to set goals, manage behavior, build relationships, and process and remember information (Jones & Kahn, 2018, p. 17). In recent years, U.S. schools have recognized the deep connection between skills, such as empathy, cooperation, and managing emotions, and traditional academic skills, and have adopted SEL programs into their curricula (Jones & Kahn, 2018, p. 18). A 2018 survey indicated that 90% of over 500 K‐12 school district administrators had invested or planned to invest in SEL products (Yettick, 2018). In comparison, therapeutic interventions are designed to help a participant to forgive a specific individual or group. In sum, forgiveness includes a skill component that forgiveness education interventions help to develop. Specific forgiveness education interventions will be elaborated on in the moderator section.

There is evidence that forgiveness education interventions reduce anger (Brouzos et al., 2019; Enright et al., 2007; Taysi & Vural, 2015) and promote forgiveness (Bonab et al., 2021; Enright et al., 2007, 2014; Freedman & Knupp, 2003; Holter et al., 2008; Park et al., 2013), even among children and adolescents who have experienced severe injustices (Freedman, 2018; Rahman et al., 2018). However, while several meta‐analytic reviews assess the efficacy of therapeutic forgiveness interventions among adult and adolescent populations (Akhtar & Barlow, 2018; Baskin & Enright, 2004; Lundahl et al., 2008; Wade et al., 2014), there has yet to be a review of forgiveness interventions created exclusively for children and adolescents (Worthington et al., 2014, p. 29). The present research synthesis constitutes an exploratory effort primarily to determine forgiveness education interventions’ effects on children and adolescent outcomes, specifically, forgiveness and anger outcomes.

## Potential moderators of forgiveness education intervention efficacy

Although forgiveness education interventions appear effective in promoting forgiveness and reducing anger, questions about moderators that affect the efficacy of forgiveness education interventions remain unaddressed. Specifically, what factors are likely to facilitate a participant's response to the intervention? Are some curriculums more effective than others? Does treatment duration play a large role in the success of an intervention? Research literature surrounding forgiveness interventions as well as school‐based social‐emotional learning interventions was reviewed to identify potential moderators. However, since this is a novel research domain, and only a small sample of studies exist, moderator analyses were conducted with an exploratory intention rather than a confirmatory intention. Therefore, no initial hypotheses regarding moderators have been identified. The following section provides a description of each potential moderator and explanation for including it in the present analysis.

### Program characteristics

Three moderators relating to program characteristics were identified.

#### Forgiveness education intervention curriculum

In the present study, all forgiveness education interventions used one of three types of curriculums. Two of the curriculum‐types follow steps outlined in explicit clinical models of forgiveness: the Pyramid Model of REACH Forgiveness (Worthington, 1998) and the Enright Process Model of Forgiveness (Enright & Fitzgibbons, 2015). Previous meta‐analyses on therapeutic forgiveness interventions have identified effect differences between the two forgiveness models (Akhtar & Barlow, 2018; Wade et al., 2014). It follows that curriculum type also may be an important moderator for forgiveness and anger because those curriculums differ by clinical model and, by extension, both the theoretical definition of forgiveness and individual lesson objectives. Additionally, it is important to recognize that curriculums present different aims. While all curriculums aim to teach a participant about forgiveness, some also aim to help participants to forgive a person or group that has offended them (like the Enright process‐based curriculum and the REACH curriculum). An overview of each of the three forgiveness education intervention curriculum types is provided below.

##### Enright story‐based curriculum

Enright and the International Forgiveness Institute Inc. published story‐based curriculum guides spanning pre‐K through 12. The curriculum uses stories to teach about forgiveness and other related moral virtues and equips children with the knowledge of how to forgive a specific person who offends if they choose to do so. Lessons begin by educating participants about the five concepts that underlay forgiveness: inherent worth, kindness, respect, generosity, and *agape* love (Enright, & Knutson, 2010). For example, participants are encouraged to perceive the person who offends from a different perspective using a moral principle called inherent worth, which is the understanding that all people are unique and have value (Enright & Fitzgibbons, 2015). During the program, participants read and discuss several age and culture‐appropriate stories that display forgiveness between characters such as *The Tale of Despereaux* by Kate DiCamillo and *Horton Hears a Who!* By Dr. Seuss (Enright & Fitzgibbons, 2015).

In this curriculum, the definition of forgiveness is presented to participants as follows: “When unjustly hurt by another, we forgive when we begin to see the inherent worth of the one who offended, then willfully abandon our right to resentment toward the person, and try to offer the wrongdoer respect, kindness, generosity, and even love” (Enright & Knutson, 2010). This forgiveness definition highlights the goal of positive actions, as well as a change in both cognition and emotions, toward the person who offends.

##### Enright process‐based curriculum

A second group of child and adolescent forgiveness intervention curriculums adhere strictly to the Enright Process Model of Forgiveness (Enright & Fitzgibbons, 2015). In these curriculums, the theoretical definition of forgiveness is the same as in Enright story‐based curriculum. Unlike the story‐based curriculums, process‐based curriculums do not have standard lesson content, such as teaching the five ingredients of forgiveness through stories. These programs are created by many different researchers who vary in how they adapt lessons that follow the process model units. The commonality across these studies is adherence to the 20 units of this model. In the Process Model, there are four phases in which the 20 units are divided. Units 1–8 address the Uncovering Phase in which the individual gains insight into whether and how the injustice and subsequent injury has compromised his or her life. Units 9–11 represent the Decisions Phase in which the individual gains an accurate understanding of the nature of forgiveness and decides to commit to forgiving based on this understanding. Units 12–15 represent the Work Phase in which the individual gains a cognitive understanding of the person who offends and begins to view the person in a new light, resulting in change in affect about the person, the self, and the relationship. The Deepening Phase includes units 16–20 and it is during these units that the individual finds increased meaning in suffering; feels more connected with others; and experiences decreased negative affect. The goal of the curriculum is to guide participants going through the process of forgiving another person, which is reflected in learning objectives of each lesson that are aligned with the 20 units or four phases of the forgiveness process.

##### REACH curriculum

The final group of intervention curriculums use Worthington's REACH model to teach children about forgiveness (Beck, 2005; Shechtman et al., 2009). Like the process‐based curriculums, the aim of REACH‐based curriculums is to help the child or adolescent to forgive someone or a group who has offended them. Worthington (2005, p. 15) defines forgiveness as two distinct types: (1) Decisional forgiveness is an implicit or explicit decision not to seek revenge and (2) Emotional forgiveness is replacing negative unforgiving emotions (i.e., resentment, bitterness, hate, hostility, anger, and fear) with positive ones (i.e., empathy, sympathy, compassion, and love). Due to this division of forgiveness into two types, studies using REACH‐based curriculums often choose measures for one forgiveness type or the other. Worthington's REACH model consists of five steps as presented in the REACH acronym: “(1) Recalling the hurt; (2) Empathizing with the one who hurt you; (3) Altruistic giving of the gift of forgiveness; (4) Committing to forgive; (5) Holding on to the forgiveness” (Worthington, 1998). Researchers who based their curriculums on the REACH model treat each step in the model as a learning objective with related sub‐objectives and class activities. For example, Shechtman et al. (2009) divided “Step 3: Giving an altruistic gift of forgiveness” into three lessons: (1) acknowledging the difficulties of forgiveness, (2) recalling a situation in which the student felt gratitude because of another's forgiveness, and (3) symbolizing the giving of an altruistic gift (i.e., a positive thought, feeling or experience related to a person who offended).

Few forgiveness interventions for children and adolescents have been based on the Pyramid Model of REACH Forgiveness since the REACH forgiveness education intervention is more widely administered to college‐age emerging adults dealing with romantic betrayals, abuses, and family problems (Shechtman et al., 2009, p. 419; Wade & Worthington, 2005, p. 163).

#### Program duration

Program duration refers to the number of sessions or lessons in a program. One of the most well‐established moderators of psychotherapy treatments is treatment duration (Howard et al., 1986). Meta‐analyses of forgiveness interventions have strongly supported that an increase in sessions increases intervention efficacy (Akhtar & Barlow, 2016; Wade et al., 2014). Additional educational research supports that learning a new task is more successful when practice is distributed over a longer period of time rather than massed into fewer long practices (Donovan & Radosevich, 1999). Thus, it is possible that forgiveness education interventions will lead to more successful outcomes if they are taught to participants over longer periods of time.

#### Instructor type

Instructor type refers to whether the forgiveness education intervention was facilitated by a teacher, a school counselor, or by a researcher. Previous SEL meta‐analyses have looked at facilitator type as a moderator (Polanin et al., 2012; Durlak et al., 2011). Polanin et al. (2012) found significantly greater treatment effects when a bullying prevention program was implemented by someone other than the teacher, such as a researcher.

### Participant characteristics

Six moderators relating to participant characteristics were identified.

#### Age and grade

A developmental trajectory of executive functions influences a child's ability to forgive. van der Wal et al. (2014) hypothesized that a child who can resist the urge to retaliate after an offense can contemplate a more thoughtful response. van der Wal et al. (2014) also reported that greater executive function control, measured by cognitive control tasks (i.e., Flanker task, go/no go), is related to a child's likeliness to forgive. The relationship between executive functions and forgiveness is further supported among a college‐age sample (Pronk et al., 2010). Other developmental aspects are also associated with forgiveness. For example, the cognitive development of concrete operations will enable children to understand the causes and consequences of people's actions (including unjust actions). Thus, children entering the preoperational stage (ages 2–7), who are still developing cognitive competencies like cooperation and understanding others’ perspectives, may have more difficulty than older peers in understanding and internalizing the value of forgiveness in relationships (Carpendale, 2000, p. 194).

#### Income level

Income level refers to whether the participants attending the group program are located in an economically disadvantaged or non‐disadvantaged area, determined by the national median income. There is some evidence to suggest that social‐emotional learning programs have been less effective in promoting social competence within high‐poverty schools (Bierman et al., 2010). However, Taylor et al. (2017) did not find a significant difference between economically diverse groups of students in their meta‐analysis of social‐emotional learning programs.

#### Offense severity

Offense severity refers to the degree to which an offense is more difficult to overcome. In a meta‐analysis on forgiveness interventions, Wade et al. (2014) found offense severity positively correlated with the outcome forgiveness. They theorized that individuals who were more severely offended may have had more room to change in terms of forgiveness (Wade et al., 2014). However, it has yet to be determined if offense severity moderates the relationship between the forgiveness education intervention and the development of forgiveness or reduction of anger. Research supports that it is more difficult to forgive a more severe offense (Fincham et al., 2005; Ohbuchi et al., 1989). Longer‐lasting transgressions appear to take a longer period to forgive than minor offenses (McCullough et al., 1998).

#### Gender

Few studies have examined gender differences related to the effectiveness of forgiveness education interventions. Bonab et al. (2021) found that eighth‐grade males benefited significantly more than females from a forgiveness education intervention with regard to forgiveness. However, there were no significant differences in anger measures between male and female participants. In addition, Beck (2005) examined gender differences among students in grades nine to twelve. Beck found that among those who participated in the forgiveness education intervention, girls had significantly more gains in forgiveness than boys. Despite these mixed results, research generally supports that females are more forgiving than males. A meta‐analysis exploring gender and forgiveness among adults in 70 studies found that women were slightly (“1/4 of standard deviation”) more forgiving than men (Miller et al., 2008). Forgiveness studies with child participants (Javed et al., 2018; Lukasik, 2000) echo this finding.

#### World zone

No studies have explored the differences in the effectiveness of forgiveness education or SEL interventions among world zones. A meta‐analytic review of cultural differences in forgiveness and revenge among adults from 16 countries (Lennon, 2013) did not find significant differences in forgiveness and revenge on four cultural dimensions. The dimensions included: individualism‐collectivism, masculinity‐femininity (gender role differentiation), low versus high power distance (acceptance of individual power inequalities), and low versus high uncertainty avoidance (desire for rules and structure). Thus, the world zone is purely an exploratory moderator.

## Purpose

The present research synthesis was an exploratory effort to investigate the effects of forgiveness education interventions aimed at increasing forgiveness and reducing anger in children and adolescents. This review sought to expand the literature for this novel research domain by meta‐analyzing 20 intervention studies from 11 countries in order to investigate the extent to which forgiveness education interventions promoted forgiveness and decreased anger among children and adolescents. In addition, program characteristics and participant characteristics that may affect forgiveness education intervention outcomes were explored. Thus, the present study addressed the following three research questions: Do forgiveness education interventions significantly increase the forgiveness in children and adolescents? Do forgiveness education interventions significantly reduce the anger in children and adolescents? What program or participant characteristics contribute to the efficacy of forgiveness education interventions?

## METHOD

### Criteria for considering studies for review

This meta‐analytic review was based on 20 published and unpublished randomized intervention studies of forgiveness education. Since this is the first review of forgiveness education interventions, researchers were tasked with operationalizing the definition of a forgiveness education intervention. To be included in this review, forgiveness education interventions had to (a) be designed for a child or adolescent population (elementary‐high school), (b) be administered in a class or a group, (c) be established as a curriculum with lesson plans, and (d) be instructed by someone rather than self‐facilitated. In addition, all studies were required to have a quantitative measure either of forgiveness or anger outcomes since these were the outcomes of interest. To mitigate the risk of bias, studies that did not randomly assign participants or classrooms into treatment and comparison conditions, and studies that did not include a comparison condition, were excluded from this synthesis.

### Outcomes

Forgiveness education interventions are thought to have direct effects on forgiveness and anger outcomes and indirect effects on several other psychological outcomes associated with relational transgressions (i.e., self‐esteem, depression, etc.). Thus, only studies that reported forgiveness or anger outcomes were included in the present research synthesis. All outcomes were reported using group means and standard deviations and so it was not necessary to convert any effect sizes (ESs). All instruments are reported in Table 1. In addition, all outcome measures are described in Appendix C.

**TABLE 1:** Characteristics of studies in the meta‐analysis

Study (*k* = 20)	Percent female	Mean grade	Location	Sessions	Curriculum	Treatment (*n*)	Comparison (*n*), group type
Hepp‐Dax (1996)	55%	5th	United States	8	Enright process‐based	11	12, Placebo
Freedman and Knupp (2003)	60%	8th	United States	8	Enright process‐based	5	5, No‐Tx control
LaTurner (2006)	51%	7th	United States	3	Enright process‐based	41	49, No‐Tx control
Beck (2005)	61%	11th	United States	6	REACH model	38	38, No‐Tx control
Enright et al. study 1 (2007)	36%	1st	Northern Ireland	17	Enright story‐based	36	57, No‐Tx control
Enright et al. study 2 (2007)	46%	3rd	Northern Ireland	15	Enright story‐based	35	47, No‐Tx control
Lin and Wu (2008)	50%	6th	Taiwan	16	Enright process‐based	38	38, No‐Tx control
Holter et al. study 1 (2008)	47%	1st	United States	17	Enright story‐based	75	44, No‐Tx control
Holter et al. study 2 (2008)	64%	3rd	United States	15	Enright story‐based	36	42, No‐Tx control
Holter et al. study 3 (2008)	50%	5th	United States	15	Enright story‐based	40	39, No‐Tx control
Shechtman et al. (2009)	51%	9th	Israel	12	REACH model	65	81, Placebo
Hui and Chau (2009)	43%	6th	Hong Kong	8	Enright process‐based	28	28, Alt Tx
Lin (2011)	50%	6th	Taiwan	24	Enright process‐based	22	22, Alt Tx
Park et al. (2013)	100%	7th	South Korea	12	Enright story‐based	16	16, No‐Tx control
Taysi and Vural (2015)	39%	4th	Turkey	10	Enright story‐based	71	47, No‐Tx control
Rahman et al. (2018)	100%	5th	Pakistan	16	Enright story‐based	4	4, Alt Tx
Yang and Chen (2017)	44%	5th	Taiwan	8	Enright process‐based	27	30, Placebo
Freedman (2018)	90%	11th	United States	31	Enright process‐based	10	11, Alt Tx
Vassilopoulos et al. (2020)	43%	6th	Greece	6	Enright‐process based	21	21, No‐Tx control
Bonab et al. (2021)	50%	8th	Iran	15	Enright story‐based	123	101, No‐Tx control

Study (*k* = 20)	Instructor	Disadvantaged area	Severity of offense	Forgiveness instrument	Anger instrument
Hepp‐Dax (1996)	Researcher	Y	Low	EFI‐C	
Freedman and Knupp (2003)	Researcher	N	Low	EFI	
LaTurner (2006)	Researcher	N	NA	Heartland and Self‐developed	Vengeance scale
Beck (2005)	Researcher	N	Low	EFI	Modified Anger scale
Enright et al. study 1 (2007)	Teacher	Y	Low		Beck Anger‐Youth
Enright et al. study 2 (2007)	Teacher	Y	Low	EFI‐C	Beck Anger‐Youth
Lin and Wu (2008)	Researcher	N	NA	EFI	
Holter et al. study 1 (2008)	Teacher	Y	Low		Beck Anger‐Youth
Holter et al. study 2 (2008)	Teacher	Y	Low		Beck Anger‐Youth
Holter et al. study 3 (2008)	Teacher	Y	Low		Beck Anger‐Youth
Shechtman et al. (2009)	School counselor	N	Low		TRIM: avoidance & revenge
Hui and Chau (2009)	Teacher	N	Low	EFI & 1 item	
Lin (2011)	Researcher	N	NA	EFI	
Park et al. (2013)	Researcher	Y	High	EFI‐C	State‐Trait Anger
Taysi and Vural (2015)	Teacher	Y	NA	EFI‐C	Beck‐Anger Youth
Rahman et al. (2018)	Researcher	Y	High	EFI‐C	Self‐developed
Yang and Chen (2017)	Researcher	N	NA	EFI	
Freedman (2018)	Researcher	Y	High	EFI	
Vassilopoulos et al. (2020)	Researcher	Y	NA	EFI‐C	Anger Expression Scale for Children—Trait
Bonab et al. (2021)	Teacher	N	Low	EFI	State‐Trait & Anger Expression

Note

“Alt Tx” refers to an alternative treatment comparison group and “No‐Tx” refers to no treatment comparison group. Among forgiveness instruments, “EFI‐C” refers to the Enright Forgiveness Inventory child version and “1 item” refers to a single validation question regarding the participant's forgiveness.

### Search strategy

**FIGURE 1:**
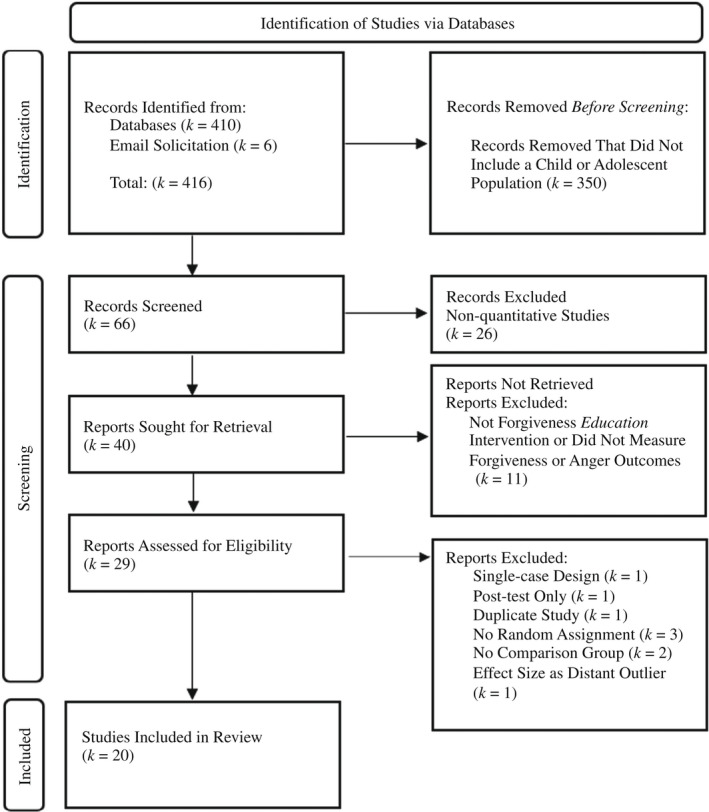
PRISMA‐style flow diagram of studies included in the meta‐analysis

Figure 1 displays the numbers of found eligible and ineligible studies. In September 2021, the following areas were searched to locate studies for inclusion: (a) an electronic search in Google Scholar, PsycINFO, and Educational Resources Information Center (ERIC) using keyword combinations. Each forgiveness term was paired with the truncated “forgiv*” to capture articles using variations on the word forgiveness, such as forgiving or forgive. The intervention search terms used included: “intervention,” “education,” “program,” “therapy,” the truncated “educat*” (i.e., education, educational, etc.), and “therap*” (i.e., therapeutic or therapies); (b) a manual search of references listed in all located studies; and (c) contacting known forgiveness researchers for unpublished studies. This report did not limit the date of publication of the studies, which span the years from 1996 to 2021. The search retrieved 410 studies and six studies were identified by forgiveness researchers prior to the search. Only 66 of those studies included a child or adolescent population which is a testament to the lack of research on this. Of those 66 studies, 40 were quantitative studies. Of the 40 quantitative studies, 11 studies either were not forgiveness education interventions or did not address the research question regarding forgiveness or anger outcomes. Of the remaining 29 studies, one was a single‐case design and thus could not produce a single post‐test outcome measure (Kim, 2016), one only measured forgiveness at post‐test (Hui & Ho, 2004), and one was a duplicate study that was available in both article and dissertation forms (Park et al., 2013). Three studies were excluded because they did not have a random assignment (Brouzos et al., 2019; Owens, 2018; Sánchez‐Hernández et al., 2021). In addition, two studies were excluded because they did not have a comparison condition, such as a control group or an alternative treatment group (Ahn‐Im, 2016; Enright et al., 2014). One study (Gambaro et al., 2008) did not contribute to the final analysis because it was considered as a distant outlier as the lower bound of the 95% confidence interval of the forgiveness effect (*g* = 8.64, CI [5.03, 12.26]) was beyond the higher bound of the pooled effect confidence interval (*g* = 0.54, 95% CI [0.36, 0.73]). Analyses with the inclusion of Gambaro et al. (2008) can be found in Appendix D. Thus, the present research synthesis included 20 total studies. No geographical or cultural restrictions were applied to this research synthesis. Studies published in traditional Chinese (Lin, 2011; Lin & Wu, 2008; Yang & Chen, 2017) were translated and included.

### Study and participant characteristics

Table 1 displays a full list of the 20 studies included in this research synthesis. Of the 1472 children and adolescents among all studies, approximately 51% were female. The mean participant age of the 20 studies was 11.66 years old (average grade 5.7), and age averages among the studies ranged from 6.5 to 17.3 years. Grade levels for each study were converted to U.S. grade equivalents. For example, primary 7 in Northern Ireland would be displayed as grade 5 in Table 1.

The studies reviewed encompassed ten countries (studies: 40% North American, 25% East Asian, 20% Middle Eastern, 15% European). Thus, samples were demographically diverse from a wide range of cultural contexts. Additionally, several studies highlighted the cultural context in which they were researching the effects of a forgiveness education intervention. Shechtman et al. (2009) implemented a forgiveness education intervention with Arab adolescents in Israel, and measured group climate (relating to Jew and Arab relations) at the conclusion of the study. Similarly, Bonab et al. (2021) measured ethnic prejudice before and after the forgiveness education intervention among eighth‐grade students in three provinces of Iran: Tehran (Persian), Eastern Azerbaijan (Azeri), and Kurdistan (Kurdish). Enright et al. (2007) supported multiple forgiveness education interventions in schools in Belfast's central city, an “interface area” characterized by Catholic and Protestant neighborhoods being near one another. Additionally, two studies point out that interventions took place in elementary schools in urban areas including Brooklyn, New York, United States (Hepp‐Dax, 1996) and Central City Milwaukee, United States (Holter et al., 2008).

Several studies highlighted special student populations. In their respective studies, Park et al. (2013) and Beck (2005) recruited adolescents who had been identified as aggressive victims. Freedman (2018) implemented her intervention in an alternative school with students who were at risk of dropping out of school. Freedman and Knupp (2003) administered a forgiveness education intervention with adolescents who had experienced parental divorce. Taysi and Vural (2015) studied fourth‐grade students in elementary schools in Isparta, Turkey, of which approximately half were from lower‐income households. Lastly, Rahman et al. (2018) reported on a unique forgiveness education intervention study in which female participants (*M*_age_ = 11.5) had been removed from abusive situations and were living in the ChildProtection and Welfare Bureau in Lahore, Pakistan. In this study, participants had received individual counseling prior to starting thegroup forgiveness education intervention. The remaining six studies did not elaborate on a special context or population, but rather, worked with typical school children.

### Coding reliability and agreement

For each study, the first and second authors of this paper documented codes for study characteristics, participant characteristics, and program characteristics. A coding manual guided the coding process (Please refer to the forgiveness education Meta Coding Sheet and the Effect Size Coding Sheet in Appendix A, as well as the Coding Manual in Appendix B). Authors were contacted for clarifications on missing data. To support that the coding is replicable and valid, both coder reliability and coder agreement were computed. For categorical variables, coder agreement was determined by Cohen's kappa, or the proportion of agreement corrected for chance. Severity of offense had the lowest interrater reliability with a kappa of .78. The kappa for income level was .83. The kappa for all other categorical moderators, including publication status, comparison group type, instructor type, curriculum type, etc., was 1.00. For continuous variables, coder reliability was determined by computing the intraclass correlation coefficient (ICC). With the exception of the session length in hours (ICC = .98), the interrater reliability of other continuous moderators (including mean age, mean grade, number of sessions) was 1.00.

### Computing ESs

The basic unit of analysis in meta‐analytic procedures is the ES. The ES for group comparisons is Cohen's *d* or the standardized mean difference. In the equation, the difference between the treatment group mean (M1) and the control group mean (M2) are transformed into standardized units for easier comparison between studies.

$$d=\frac{\left(M1‐M2\right)}{SD_{\text{pooled}}}.$$

However, Cohen's *d* tends to overestimate the absolute value of the population parameter in small samples (Borenstein et al., 2009, p. 50). Using the methods outlined by Borenstein et al. (2009, p. 27), the unbiased ES Hedges’ *g* was derived by multiplying *d* by a correction factor (*J*). An approximation of the formula for *J* is as follows:

$$J=1‐\frac{3}{4df‐1}.$$

Thus, Hedges’ *g* (Hedges, 1981) was the ES used in this research synthesis. Moving forward, Hedges’ *g* will be abbreviated as “*g*” in this review. More weight was given to the results from studies that had larger sample sizes by weighting each ES estimate by the reciprocal of its variance (Borenstein et al., 2009, p. 65).

Some studies contained multiple measures for the outcome of interest (i.e., forgiveness and anger). Multiple ESs of the same outcome measure were aggregated to produce one ES per study. The R package “MAd” was used to automate the ES computation and aggregation process (Del Re & Hoyt, 2010). The R package “metafor” (Viechtbauer, 2010) was used to conduct the omnibus analyses, heterogeneity tests, and moderator analyses.

### Random effects model and general analyses

Effect sizes were treated as random effects in all analyses based on the assumption that there were systematic differences among studies beyond sampling error (Hedges & Vevea, 1998). $\tau^{2}$ (tau‐squared), the between‐studies variance, or the amount of true heterogeneity, was calculated and accounted for as a source of error in the random effects model. If the true ES of all studies (across an infinite number of studies) was known, the variance would be $\tau^{2}$. $\tau^{2}$ was estimated using the default estimation within the Metafor package in R which is called “REML” or restricted maximum likelihood estimator.

A heterogeneity test (*Q*‐statistic) determined whether the variability among the sample of ESs is greater than would be expected by sampling error alone (Rosenthal et al., 2006). *I* ^2^ represents the percentage of systematic (non‐chance) variance. Procedurally, after running the fixed‐effects model in R, high heterogeneity (*I* ^2^ ≥50%) assisted in the determination to use a random effects model.

### Moderator analyses

Prior to the moderator analyses, the forgiveness and anger outcomes in the same study were aggregated to derive the study's combined positive outcome effect. Moderator effects became relevant to explain some of the heterogeneity between studies. The R package “metafor” (Viechtbauer, 2010) was used to perform meta‐regressions to analyze the continuous moderators (i.e., mean age). R software allowed for estimates of the intercept (*B*_0_), slope (*B*_1_), 95% CI for *B*_1_, and the corresponding *p*‐value for each moderator. For the categorical variables, such as instructor type, a meta‐analysis is conducted for each moderator group. That is to say that the mean ES and standard error for each sub‐group of studies are computed before comparing them to see if they are significantly different from one another. The categorical moderator analysis has been likened to an ANOVA model in that the researcher is interested in comparing the group mean ES for two or more groups. Differences between levels of the categorical moderator were tested for all studies containing either anger or forgiveness outcomes. Output from R helps to easily determine the ES, 95% CI, *Q*, and *p*‐value for each group. If *p* < .05, then the moderator variable is a significant moderator of the study ES.

### Multiple comparison group types

Thirteen studies compared forgiveness education interventions to a no‐treatment control group. Seven studies compared a forgiveness intervention to a non‐specific alternative skill‐based program (*k* = 4) or a placebo program (*k* = 3). All 20 studies were included in the omnibus analyses, regardless of the type of comparison group. This decision was made based on the idea that all the comparison groups are comparable in that they do not provide a forgiveness education intervention to the participants. This method was used in a previous forgiveness therapy meta‐analysis (Lundahl et al., 2008). The comparison group type was coded as a moderator in the moderator analysis to substantiate comparison group equivalency. It was expected that there would be lower ESs among studies with alternative treatment comparison groups since it is harder for a treatment to differentiate itself from another established treatment than it is to differentiate from no treatment. However, significant ESs were found among studies with alternative treatment, placebo treatment, and control comparison groups, further supporting the decision to combine the studies into one large study sample for omnibus analyses.

### Publication bias

Publication bias was considered during the literature search and analyses. Rosenthal (1979) highlights the “file‐drawer problem,” which states that studies with small or nonsignificant ESs tended to remain unpublished. To address this potential bias, unpublished works from three dissertations (Beck, 2005; Hepp‐Dax, 1996; LaTurner, 2006) were included. In addition, Egger's regression test (Egger et al., 1997) was employed to estimate publication bias by calculating the relationship between ESs and variability in a meta‐regression model. A sensitivity analysis was conducted using Mathur and VanderWeele’s (2020) protocol, to check the severity of the publication bias.

### Further exploratory analyses

Do forgiveness education interventions contribute lasting effects on a student's forgiveness and anger outcomes? To answer this question, durability meta‐analyses were conducted. Among the seven studies that contained follow‐up data, the summary effect from baseline to follow‐up was compared to the summary effect from baseline to post‐test.

Furthermore, several studies reported mental health outcomes related to forgiveness or anger. Thus, other exploratory analyses for these outcomes were also conducted. The most common outcomes reported were depression (*k* = 7), hope (*k* = 5), self‐esteem (*k* = 4), and empathy (*k* = 3). Due to the limited number of studies that included these outcome measures, the overall effect omnibus analysis and subsequent moderator analyses did not include these outcomes.

## RESULTS

### Omnibus analyses

Because Hedges’ *g* is simply Cohen's *d* corrected for bias, Cohen’s (1988) convention for interpreting *d* can be used to interpret the present results. Cohen’s (1988) convention for interpreting *d* is as follows: *d* = 0.2 represents a small ES, *d* = 0.5 represents a medium ES, and *d* = 0.8 represents a large ES. For a large effect, for example, this means that the difference between the control and intervention groups’ means is >0.8 *SD*s, or a substantial difference. Assuming a normal distribution, this would mean that about 79% of the control group is below the mean score of the average participant in the intervention group.

### Forgiveness as outcome

**FIGURE 2:**
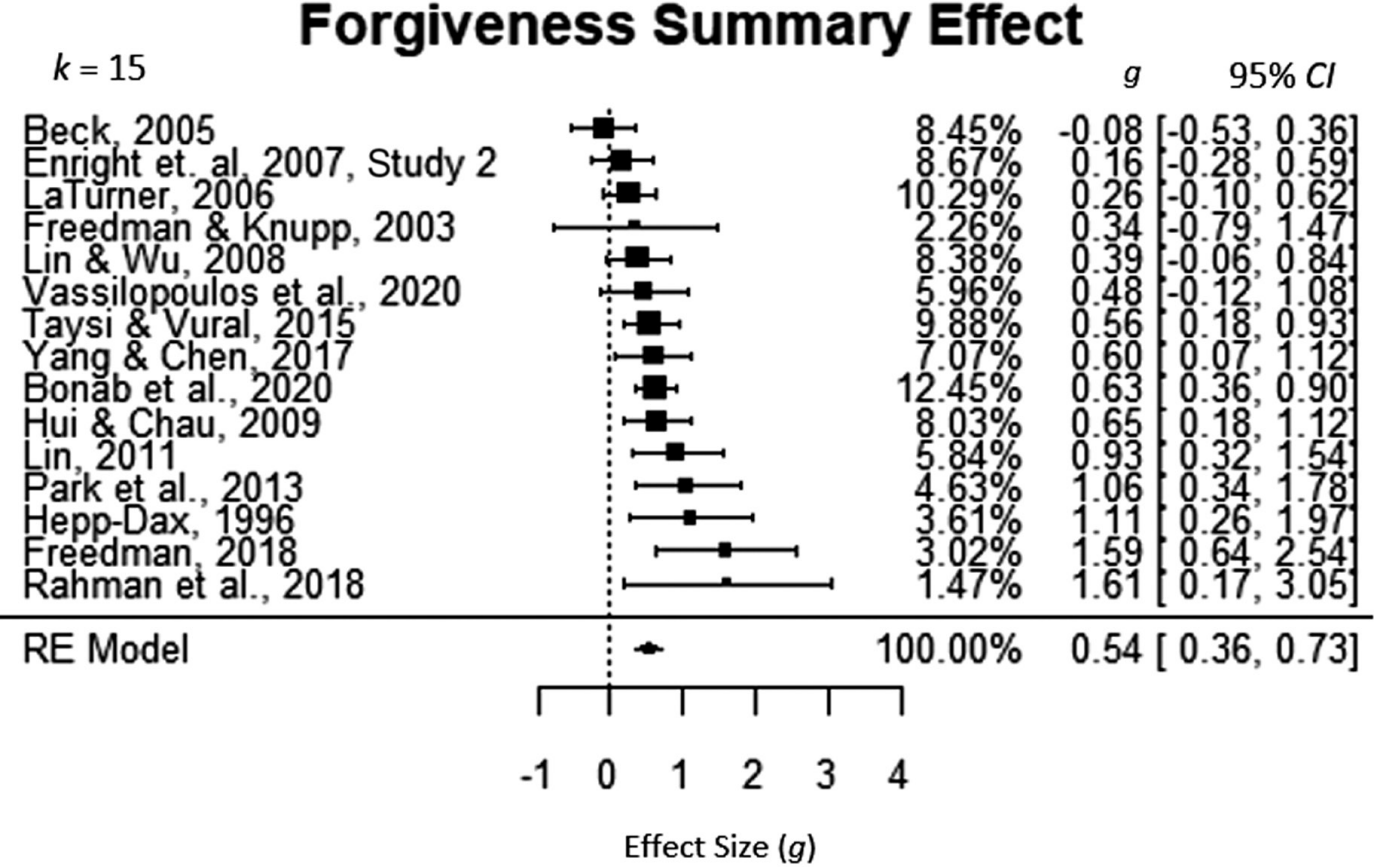
Omnibus analysis: forgiveness education intervention and forgiveness. CI, confidence interval; RE, random effects

Of the 20 studies in this research synthesis, 15 studies measured forgiveness as an outcome. Results from the forgiveness omnibus analysis displayed in Figure 2 suggested that forgiveness education interventions have a significant positive medium effect on forgiveness outcomes (*g* = 0.54, 95% CI [0.36, 0.73]). Thus, the average participant in the intervention group showed greater change in forgiveness levels over the course of the intervention than about 70% of those in the no‐intervention group. The between‐study heterogeneity was significant (*Q*(14) = 26.02, *p* = .02574), so the random effects model was used in this analysis. The percent of systematic variance or *I* ^2^ is 43.99% ($\tau^{2}=.0507$).

### Anger as outcome

**FIGURE 3:**
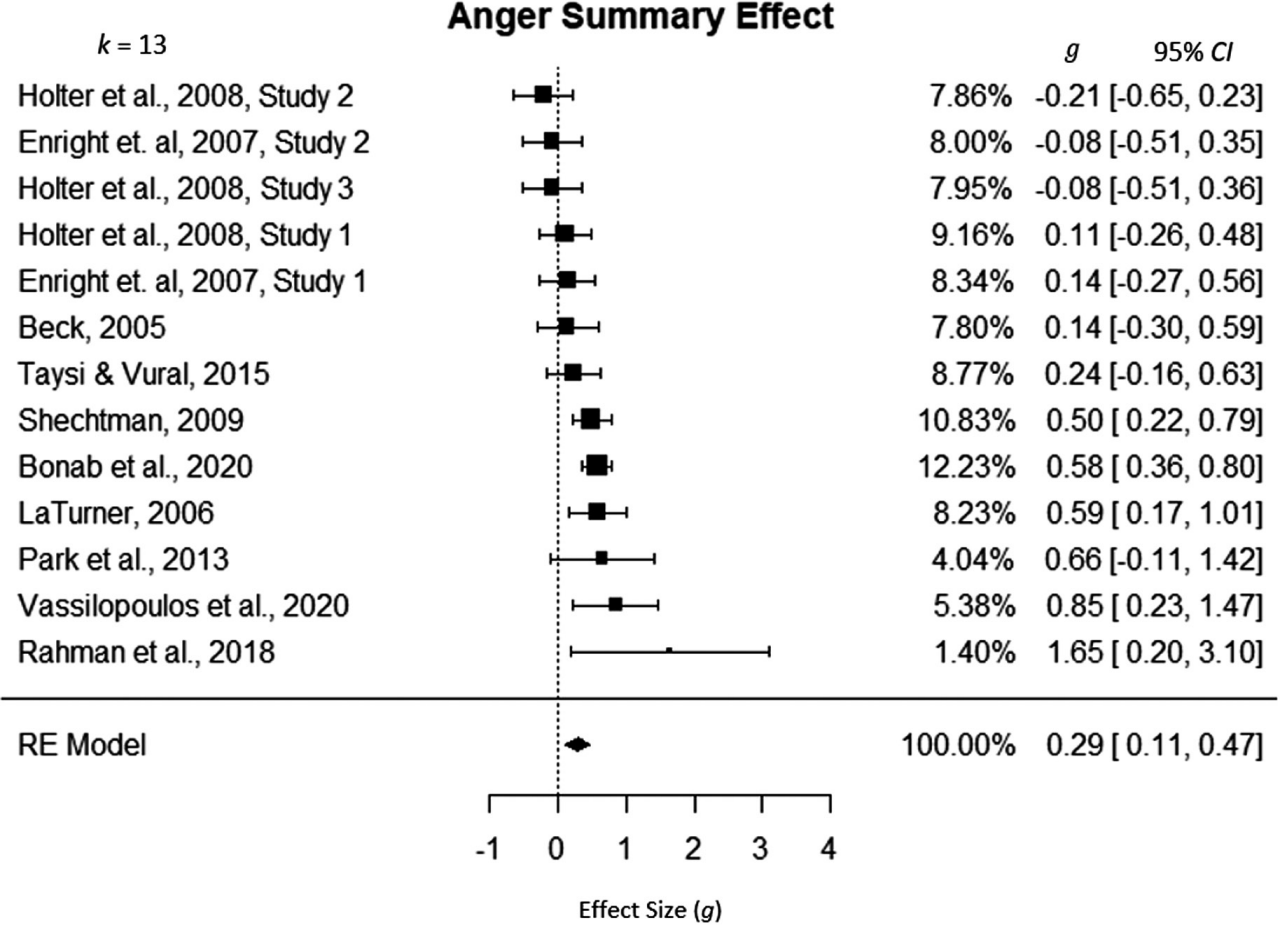
Omnibus analysis: forgiveness education intervention and anger. CI, confidence interval; RE, random effects

Thirteen studies measured anger outcomes (see Figure 3). The omnibus analysis revealed a significant small positive effect of forgiveness education interventions on anger outcomes (*g* = 0.29, 95% CI [0.11, 0.47]). This result demonstrated that the average participant in the forgiveness education intervention group experienced a greater change in anger over the course of the intervention than about 61% of those in the no‐intervention group. The between‐study heterogeneity was significant (*Q*(12) = 29.89, *p* = .0029), so the random effects model was used in this analysis. The large amount of heterogeneity in ESs (*I* ^2^ = 57.14%, $\tau^{2}=.0566$) suggests variance among studies beyond what is expected due to sampling error.

### Follow‐up data

Six studies provided follow‐up data for forgiveness outcomes. Follow‐up times ranged from 4 weeks to 1 year after post‐test and averaged about four months (in weeks, $\bar{x}$ = 16.66, *SD* = 16.23). The baseline to post‐test forgiveness summary effect was medium, *g* = 0.64, 95% CI [0.46, 0.82]. Similarly, the baseline to follow‐up summary effect was also medium (*g* = 0.62, 95% CI [0.34, 0.92]). Thus, the average intervention group participant maintained greater forgiveness scores than the average no‐intervention group participant over the follow‐up period. However, there was no significant difference in the change between the no‐intervention and intervention groups’ means from post‐test to follow‐up.

Five studies provided follow‐up data for anger outcomes. Follow‐up times ranged from 8 weeks to 1 year after post‐test and averaged about five months (in weeks, $\bar{x}$ = 22.6, *SD* = 17.29). Among the five studies, the baseline to post‐test summary effect for anger was medium (*g* = 0.52, 95% CI [0.36, 0.67]). In comparison, the baseline to follow‐up summary effect for anger was medium to large (*g* = 0.76, 95% CI [0.54, 0.99]). From baseline to follow‐up, participants in the forgiveness education intervention group experienced a greater change in self‐reported anger from baseline to follow‐up than over 77% of those in the no‐intervention group. Overall, the two results suggest that the difference in anger scores between the no‐intervention and intervention groups’ means became more distinct from post‐test to follow‐up by an increase of about 0.24 *SD*s.

### All other outcomes

In addition to reporting forgiveness and anger outcomes, some studies also included measures associated with forgiveness and anger that were not directly targeted by the intervention. The outcomes included depression, hope, self‐esteem, and empathy. Each outcome's summary effect and confidence interval are presented in Table 2. Empathy was the only outcome ES that was statistically significant with a small to medium effect (*g* = 0.32, 95% CI [0.008, 0.64]). Thus, among those three studies’ samples, the average participant in the forgiveness education intervention group experienced a greater change in empathy over the course of the intervention than over 61% of those in the no‐intervention group. However, because few studies measured empathy as an outcome, it is important to further explore this variable, in future forgiveness education interventions.

**TABLE 2:** Omnibus effect sizes and heterogeneity tests for additional outcomes

	*k*	*g*	95% CI	*Q*	*p*	*I* ^2^
Depression	7	0.11	[−0.23, 0.45]	17.45	.52	68.20%
Hope	5	1.70	[−0.09, 3.49]	22.62	.06	96.25%
Self‐esteem	4	0.02	[−0.39, 0.43]	3.25	.92	14.66%
Empathy	3	0.32	[0.008, 0.64]	2.55	.04	28.18%

Note

Studies were modeled as random effects, *k* = number of studies, *g* = effect size (Hedges’ *g*; Hedges, 1981); *Q* = homogeneity test; *p* = probability value for *Q* statistic under *H*_0_ (*df* = *k* − 1); *I* ^2^ = percentage of variance in effect sizes that is attributable to systematic variation.

### Overall effectiveness of forgiveness education interventions

To evaluate the overall efficacy of forgiveness education interventions of the 20 intervention studies, forgiveness and anger outcomes were combined. The summary effect was medium (*g* = 0.41, 95% CI [0.26, 0.57]). The result demonstrated that, on average, participants of forgiveness education interventions experienced positive outcomes relating to the reduction of anger and growth of forgiveness when compared with participants who did not receive a forgiveness education intervention. Taken another way, the average participant in the forgiveness education intervention group experienced a greater overall change in outcomes than 65% of those in the no‐intervention group. The between‐study heterogeneity was significant (*Q*(19) = 46.16, *p* = .00047), so the random effects model was used in this analysis. The results from the combined omnibus analysis indicated substantial heterogeneity ($\tau^{2}=.2617$, *I* ^2^ = 59.34%). Moderator analyses were conducted to further account for heterogeneity between studies and to investigate the third exploratory research question, “What program or participant characteristics contribute to the efficacy of a forgiveness education intervention?”.

### Moderator analyses

#### Moderators of program characteristics

Three moderators related to program characteristics were explored. The first, duration, was coded in three ways: number of sessions, sessions per week (frequency), and total amount of time (hours). All studies reported the number and distribution of sessions (i.e., weekly, everyday). Three studies did not indicate the total amount of time in a lesson. The remaining studies (*k* = 17) contained sessions ranging from 45‐ to 120‐min sessions. The number of sessions in a program and total hours were not statistically significant predictors of treatment efficacy (see Table 3). However, sessions per week was a statistically significant predictor of treatment efficacy.

**TABLE 3:** Meta‐regression analysis of combined outcome effect sizes

	*k*	*B*_0_	*B*_1_	95% CI (*B*_1_)	*z* (*B*_1_)	*p*
Number of sessions	20	0.27	0.01	[−0.018, 0.042]	0.80	.43
Sessions per week	20	0.11	0.21	[0.056, 0.354]	2.70	.007**
Total hours in program	17	0.20	0.02	[−0.020, 0.055]	0.94	.35
Mean age	20	−0.30	0.06	[0.010, 0.110]	2.33	.02*
Mean grade	20	0.11	0.05	[−0.002, 0.106]	1.89	.06
Gender	20	−0.15	0.01	[−0.0004, 0.02]	1.89	.06

Note

Univariate analyses used a mixed model (studies random, levels of moderator variables fixed); *k* = number of studies, *B*_0_ = intercept; *B*_1_ = slope; *z* (*B*_1_) = *z* statistic for *B*_1_; CI = confidence interval.

* *p* < .05.

** *p* < .001.

Second, it was found that among this 20‐study sample, forgiveness education interventions are significantly effective whether they are facilitated by schoolteachers or by researchers (see Table 4). However, the ES of the subgroup containing interventions instructed by researchers was medium to large (k=11,g=0.62) and the ES of the subgroup of interventions facilitated by teachers or school counselors was small to medium (k=9,g=0.26). Considering the subgroup of eleven interventions facilitated by researchers, the average participant in the intervention group experienced a greater change in forgiveness and anger scores than over 72% of those in the no‐intervention group.

**TABLE 4:** Single‐moderator analyses—categorical moderators

	*K*	*g*	95% CI	*Q*	*p*	*I* ^2^
*Program characteristics*						
**Instructor type**						
Teacher/school counselor	9	0.26	[0.05, 0.47]	22.59	.014	65%
Researcher	11	0.62	[0.38, 0.86]	20.25	.000	51%
**Curriculum**						
Enright story‐based	9	0.27	[0.04, 0.49]	26.04	.020	69%
Enright process model‐based	9	0.66	[0.40, 0.92]	9.60	.000	17%
REACH model‐based	2	0.29	[−0.14, 0.72]	3.71	.188	73%
*Participant characteristics*						
**Academic divisions**						
1–3	4	0.03	[−0.25, 0.30]	1.58	.842	0%
4–5	5	0.45	[0.15, 0.75]	11.50	.003	65%
6–8	8	0.58	[0.37, 0.80]	4.50	.000	0%
9–12	3	0.44	[0.10, 0.78]	10.02	.011	80%
**Income level**						
Disadvantaged	11	0.34	[0.13, 0.56]	30.99	.002	68%
Not disadvantaged	9	0.48	[0.26, 0.70]	9.66	.000	17%
**Severity**						
Not stated	5	0.49	[0.22, 0.77]	3.03	.000	0%
Mild offense	12	0.29	[0.11, 0.47]	29.57	.001	63%
Severe offense	3	1.21	[0.65, 1.78]	2.16	.000	7%
**World zones**						
East Asian	5	0.65	[0.36, 0.95]	2.54	.000	0%
European	3	0.24	[−0.10, 0.58]	3.75	.169	47%
Middle Eastern	4	0.56	[0.28, 0.83]	3.91	.000	23%
North American	8	0.22	[−0.02, 0.45]	19.30	.007	64%
*Study characteristics*						
**Comparison group**						
No‐Tx Control	13	0.27	[0.11, 0.43]	25.77	.001	53%
Placebo	3	0.62	[0.26, 0.99]	1.80	.001	0%
Alternative‐Tx Control	4	0.98	[0.57, 1.39]	4.41	.000	32%
**Publication status**						
Published	17	0.43	[0.25, 0.60]	39.88	.000	60%
Unpublished	3	0.36	[−0. 05, 0.78]	5.55	.087	64%

Note

Univariate analyses used a mixed model (studies random, levels of moderator variables fixed); k = number of studies, Hedges’ g = effect size; C.I. = confidence interval; Q = homogeneity test; I2 = percentage of variance in effect sizes that is attributable to systematic variation.

The third program moderator explored was the curriculum type. Subgroups of forgiveness education interventions that used the Enright story‐based curriculums (*k* = 9, *g* = 0.27) and Enright process‐based curriculums (*k* = 9, *g* = 0.66) both yielded significant effects. The subgroup of interventions that used REACH model‐based curriculums was not significant (*k* = 2, *g* = 0.29), although this subgroup's ES was comparable to the Enright story‐based curriculums subgroup. Thus, this lack of significance is likely related to the small sample of REACH studies (*k* = 2) in the analysis. Including more studies that used the REACH model‐based curriculums in future analyses would elicit more reliable results.

The Enright process‐based curriculums subgroup had a medium to large effect of 0.66, the strongest ES out of the curriculum type groups. Thus, among these nine studies, the average participant in the intervention group experienced a greater change in forgiveness and anger scores than over 74% of those in the no‐intervention group.

#### Moderators of participant characteristics

Six moderators regarding participant characteristics were explored in this research synthesis. First, the mean age of study participants proved to be a significant continuous moderator of intervention efficacy. Each additional year leads to an increase of (B1) 0.06 *SD*s in treatment outcomes relative to the control group. For participants with a mean age of 16, the predicted ES would be 0.66 or a medium to large effect. In comparison, the predicted ES for a group with the mean age of eight would be 0.18 or a very small effect. The second moderator exploring participant characteristics was the mean grade. Interestingly, the mean grade was not a significant continuous moderator of treatment efficacy which suggests a misalignment between the age of the child or adolescent and the grade in which they are enrolled among the 20 studies in this research synthesis. However, the categorical moderator analysis of mean grades, divided into four subgroups, showed significant effects for all subgroups except for the early primary (grades 1–3) studies subgroup (*k* = 4, *g* = 0.03). The third exploratory moderator was gender, which was calculated as the percentage of female participants. The results indicated that a higher percentage of female participants was not a significant moderator of treatment efficacy.

The fourth moderator relating to participant characteristics that was explored was the average severity of offense experienced by study participants. Among the 15 studies that included severity of offense information, significant effects were found among studies with participants who had, on average, experienced severe offenses (*k* = 3, *g* = 1.21) and mild offenses (*k* = 12, *g* = 0.29). Within the subgroup of studies with severe offenses, the difference between the no‐intervention and intervention groups’ means is >1.21 *SD*s. Sawilowsky (2009), who supported an extension to Cohen's convention, referred to an ES of 1.2 as “very large.” However, only three studies composed this subgroup, so further exploration of the relationship between forgiveness education interventions and average offense severity among participants is highly encouraged.

The fifth moderator relating to participant characteristics was the estimated mean income level of participants. Significant effects were found among studies with participants who attended school in disadvantaged areas (*k* = 11, *g* = 0.34) and non‐disadvantaged areas (*k* = 9, *g* = 0.48).

The final moderator was the world zone. World zones were based on the geographic regions in which studies took place. Studies in Pakistan and Turkey were included in the Middle Eastern zone with Iran and Israel since both countries border Iran. Significant effects were found among studies in East Asian (*k* = 5, *g* = 0.65) and the Middle Eastern (*k* = 4, *g* = 0.56) world zones. The North American studies subgroup (*k* = 8, *g* = 0.22) and European studies subgroup (*k* = 3, *g* = 0.24) were not statistically significant.

#### Moderators of study characteristics

Moderators of study characteristics were also included to explore whether differences in study designs, among the 20 studies, contributed to the efficacy of the forgiveness education interventions. Two moderators exploring study characteristics are represented in Table 4. The first moderator to be explored was the comparison group type. The subgroups included three levels of comparison group types, including no treatment‐control, placebo, and alternative treatment comparison groups. All three subgroups yielded significant effects. The subgroup of studies that had alternative treatment comparison groups (*k* = 4) had the strongest ES (*g* = 0.98), or a large effect by Cohen's convention. Among these four studies, the average participant in the forgiveness education intervention group experienced a greater overall change in combined outcomes than over 83% of participants in an alternative treatment group. Intervention effectiveness based on publication status presented substantial differences: the published studies showed significant efficacy of forgiveness education interventions while the unpublished dissertations did not. However, only three studies composed the subgroup of unpublished interventions, and so the inclusion of more unpublished studies would strengthen this result.

### Publication bias

**FIGURE 4:**
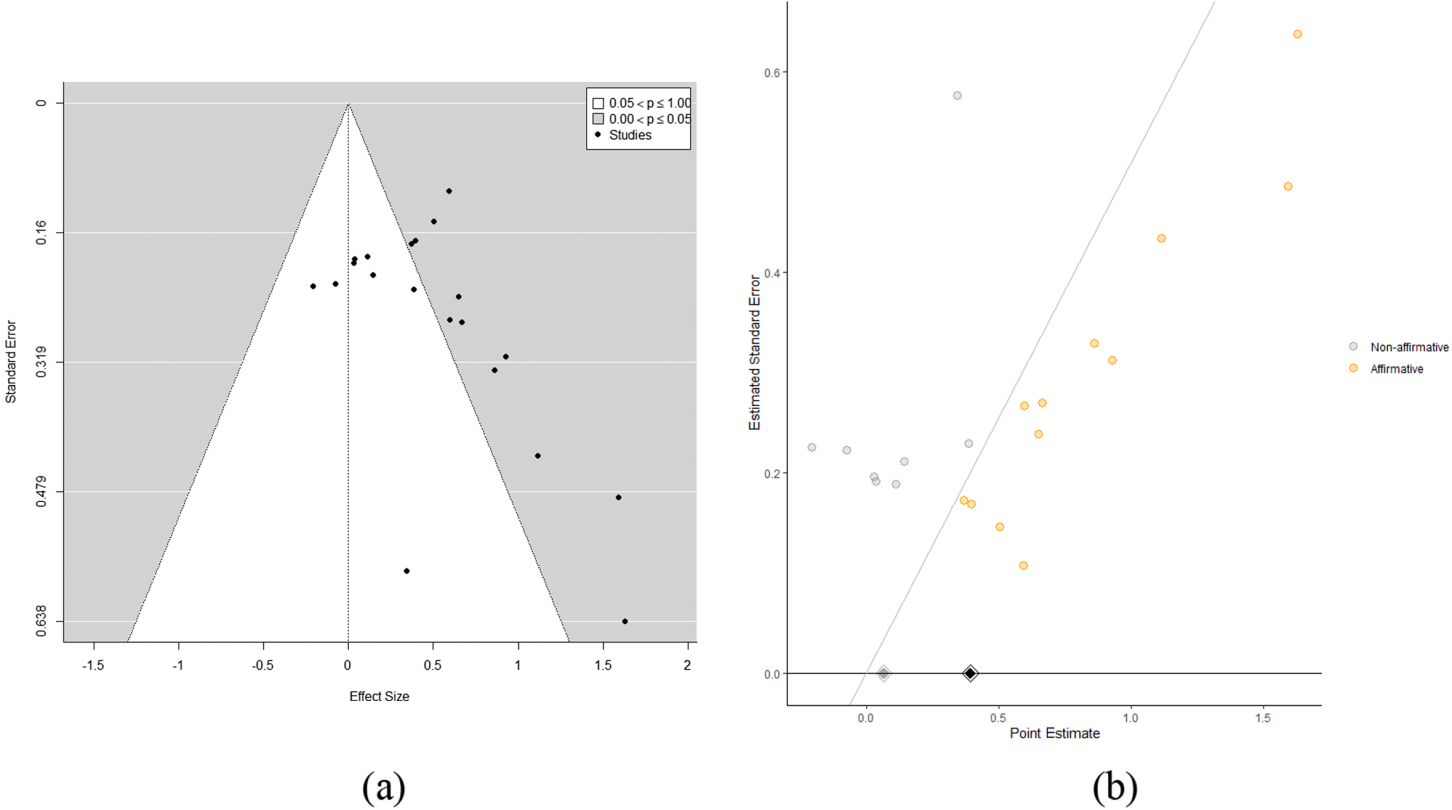
(a) Standard funnel plot versus (b) significance funnel plot for data generated with publication bias and with right‐skewed population effect sizes. Effect sizes lying on the diagonal line have exactly *p* = .05. Grey dot: non‐affirmative; orange dot: affirmative; black diamond: pooled point estimates within all studies; gray diamond: pooled point estimates within only the studies with non‐affirmative results

All ESs of pooled point estimates (of forgiveness and anger measures) are displayed in a standard funnel plot in 4a. To assess the publication bias, an extension of Egger's regression test (Egger et al., 1997; Rothstein et al., 2005) was run by calculating the weighted regression of the ESs on their standard errors, weighted by the inverse of their variances. The weighted regression slope is *β*_1_ = 0.18 (95% CI  [−0.22, 0.58]), which indicates the skewness of the funnel plot is possibly due to the publication bias (Lin & Chu, 2018). Second, we conducted a sensitivity analysis to check the severity of publication bias, which is the ratio of how much more likely “statistically significant” or affirmative results (i.e., results with a two‐tailed *p* < .05) are to be published than “nonsignificant” or non‐affirmative results (i.e., results with a two‐tailed *p* ≥ .05; Mathur & VanderWeele, 2020). Figure 4a shows right‐skewed independent ESs generated with publication bias. The significance funnel plot in Figure 4b suggests a positive correlation between the ESs and their standard errors. This approach assumes that such correlation arising from selection is due to publication bias rather than to correlation between the ESs and standard errors in the underlying population (Mathur & VanderWeele, 2020). The worst‐case estimate (represented by the gray dot in Figure 4b) from the meta‐analyses of 8 non‐affirmative pooled point estimates is *g* = 0.06 (95% CI [−0.07, 0.21]). To estimate the severity of publication bias, we attenuated the pooled point estimates to the null and to a non‐null ES of 0.1 (Mathur & VanderWeele, 2020). Under the random‐effect specification, it is impossible for the publication bias to attenuate the pooled point estimate to the null. For the publication bias to attenuate the pooled point estimate to 0.1, affirmative studies would need to be at least 27‐fold more likely to be published than non‐affirmative studies. Thus, the overall conclusion is that, regardless of the severity of publication bias, this meta‐analysis provides strong evidence for an average effect in the observed direction, albeit possibly of small size (Mathur & VanderWeele, 2020).

## DISCUSSION

The present meta‐analytic review evaluated the effects of forgiveness education interventions on child and adolescent populations. Among the 20 studies included in this research synthesis, it was found that forgiveness educations interventions contributed to positive forgiveness and anger outcomes. Results echoed findings from previous reviews of forgiveness interventions with primarily adult populations. For example, Akhtar and Barlow (2018) reported a medium effect favoring the forgiveness intervention group for reducing anger and hostility. They also reported a medium effect favoring the intervention group for forgiveness. In a meta‐analysis of therapeutic forgiveness interventions conducted by Wade et al. (2014), participants receiving interventions reported significantly greater forgiveness toward a specific offender than participants not receiving treatment. The present study suggests that children and adolescents can benefit from forgiveness education interventions.

Many of the forgiveness interventions with adults focused on clinical samples (see Reed & Enright, 2006). In other words, the samples already were selected for emotional compromise. Thus, for some adult samples, the participants had a long way to go to decrease in such variables as anger, anxiety, and depression. In contrast, participant samples for forgiveness education interventions were predominantly a non‐clinical sample of youth in school classrooms. In other words, there was the potential for less variability in the anger scores from pretest to post‐test and follow‐up test for these student samples. Nonetheless, the forgiveness education interventions did show statistical significance in the reduction of anger. In a similar way, many of the adult samples were selected for severe injustices in which people were not very forgiving, allowing for greater variance among the testing periods. The student samples in forgiveness education interventions, as in the case with anger, had a smaller potential for change, and yet statistically significant change in forgiveness was observed.

Exploratory moderator analyses also revealed several findings of interest. First, program characteristics of the forgiveness education interventions affect the intervention efficacy. It was found that interventions facilitated by teachers as well as interventions facilitated by researchers were both significantly effective. With regard to curriculum type, interventions that utilized the Enright process‐based and story‐based curriculums saw significant positive changes in anger and forgiveness when compared with the no‐intervention groups. Interventions that used a REACH model‐based curriculum (*k* = 2, *g* = 0.29) were not statistically significant even though their strength of effect was comparable to that of the interventions that used the Enright story‐based curriculums (*k* = 9, *g* = 0.27). Thus, a larger sample of studies with REACH model‐based curriculums would allow for a better exploration of the curriculum's efficacy. Regarding program duration, total number of sessions and total program hours did not significantly predict program effectiveness. This result was surprising given that previous meta‐analyses of forgiveness interventions supported that an increase in sessions increases intervention efficacy (Akhtar & Barlow, 2018; Wade et al., 2014). One possible explanation for why program duration is not a significant moderator is that studies were sufficiently long, thus reducing variability in terms of the length of the program. Among the 20 studies in the current synthesis, results suggested that forgiveness education programs of both short and long durations can lead to significant positive change in anger and forgiveness outcomes. Additionally, sessions per week was significantly predictive of program effectiveness. However, this result should be interpreted with caution since 13 of the 20 studies had only one session per week, and the full sample's standard deviation was only 1.35 sessions ($\bar{x}=1.\;7$ sessions). Therefore, it was likely that sessions per week was confounded by another variable. For example, the seven intervention studies with more than one session per week were also more likely to be facilitated by a researcher.

Participant samples varied across world nations, grades, income levels, and severity of offense experienced. The average age of the participants in an intervention predicted positive forgiveness and anger outcomes when compared with no‐intervention groups. Specifically, medium to large effects were found among late primary school‐aged participants (grades 4 to 5) through high school‐aged participants (grades 9 to 12). However, interventions among early primary‐aged participants (grades 1 to 3) resulted in a non‐significant effect when compared with the other academic divisions. Upon closer observation, the four studies done with early primary‐aged participants all exhibit the same characteristics which are supported by an *I* ^2^ or percentage of systematic (non‐chance) variance of 0%. These studies were taught by teachers as instructors; the participants experienced low severity of offense (Enright et al., 2007, study 2 does not report this), and they all used the Enright story‐based curriculum. Given this observation, it was likely that the categorical moderator of academic division was confounded by other variables. One explanation is that younger children probably do not experience as severe offenses as their older peers. Even though this may be the case, it does not mean that they cannot benefit from learning about forgiveness, taught by their teachers using a story‐based curriculum, as the improvement in increasing forgiveness and reducing anger was clearly observed among the early elementary group (Enright et al., 2007; Holter et al., 2008).

A surprising finding was that studies from East Asian and Middle Eastern world zones had moderate significant effects, while studies among North American and European zones had small nonsignificant effects. However, this finding should be viewed with reservations since zones were based on geographic region and could not account for important ethnic characteristics related to forgiveness (i.e., language, national trauma, religion, etc.). Still, this finding highlights the importance of further exploring specific ethnic trends regarding the effectiveness of forgiveness education interventions.

It is worth recognizing that forgiveness education interventions were effective regardless of whether the school was located in an economically disadvantaged or an economically advantaged area. The present sample of 20 studies supported a slightly higher effect in promoting social competence within economically disadvantaged schools which is contrary to the existing finding about other social‐emotional learning programs (Bierman et al., 2010). Furthermore, high levels of economic disadvantage among students were found to be associated with heightened student aggression (Colder et al., 2000). Forgiveness education interventions were effective in reducing students’ anger suggesting that interventions may be helpful to students in less economically advantaged schools who could see a reduction in aggressive behaviors by learning knowledge of forgiveness and practicing skills to improve social competence. Another notable finding was that the students who had experienced the most severe offenses had the largest effects in terms of forgiveness growth and anger reduction (*k* = 3, *g* = 1.21) when compared with the no treatment or alternative treatment comparison groups. The students who had experienced mild offenses also had significant growth in forgiveness and reduction in anger (*k* = 12, *g* = 0.29). Upon closer observation of these interventions’ shared characteristics, the groups with severe offenses were exclusively led by researchers (often psychologists) in smaller groups ranging from 4 to 16 participants ($\bar{x}=10$). The groups with mild offenses were led by schoolteachers in larger classroom settings. Skaar et al. (2016) advocated that the severity of offense must be considered prior to a forgiveness education intervention among a child or adolescent population. They used the existing three‐tier model in Positive Behavioral Interventions and Supports (PBIS) program as a suggestion for implementing forgiveness education (FE) interventions: Tier 1 introduces FE to everybody in a classroom; Tier 2 targets students who have more anger (identified by school counselors, teachers or through an anger assessment) and implements FE in small groups; Tier 3 helps the students who have serious issues, or have experienced a serious incident in their lives, in an intensive intervention on a one‐on‐one basis. The current class‐based FE intervention programs are similar to Tier 1 by introducing FE to everybody in the class, while the small group‐based FE intervention programs are like Tier 2 or 3. It has yet to be determined if this model of best‐fit is more effective or as practical as current group‐based FE intervention programs. The results found in this meta‐analysis provided some support that the FE intervention programs are worth implementing for all three tiers of PBIS because the significant effect was observed regardless of the severity of offenses experienced by participants.

### Limitations

This research synthesis has several limitations. The first limitation is due to the fact that forgiveness education interventions are a recent development and, therefore, the present results were based on only 20 intervention studies. Thus, results from moderator analyses could potentially fluctuate in statistical significance based on the addition or omission of a single study. Similarly, the total amount of studies included in this meta‐analysis was too few to run separate moderator analyses for each academic division. A second limitation is that the severity of offense moderator was subjectively coded. The coding manual contains definitions of mild and severe offense, and interrater reliability was strong, however, there were a few studies that contained groups of both mild and severe cases that were coded at the raters' discretions. Furthermore, five studies did not report offense data at all which is not unexpected given that the primary goal of forgiveness education interventions was to educate students about forgiveness and how to forgive instead of asking them to practice forgiveness. A third limitation is that all measures used to assess forgiveness and anger were self‐reported measures. Although self‐report measures are effective for assessing internal and subjective experiences such as forgiveness, these measures may include biases from socially desirable responding or halo effects. A fourth limitation is that the moderator analyses are only correlational in nature and cannot imply causation without further experimental investigation. A final limitation is that the reliance on published research studies might overestimate the magnitude of effect of forgiveness education interventions on forgiveness and anger outcomes.

### Future policy and research implications

As the need for social‐emotional learning programs in schools increases (Yettick, 2018), this meta‐analytic review offers educators another effective option for helping students learn to deal with their emotions, work through interpersonal conflict, and heal from deep hurt, all much needed skills for positive emotional and psychological well‐being. The present review of forgiveness education interventions was also useful for identifying gaps in research and recommendations for studies. First, studies should be carried out to compare the effectiveness among different curriculums of forgiveness education interventions as well as to compare the effectiveness between these curriculums and other widely used social‐emotional learning curriculums. Second, variables, such as type of instructor and severity of the offense experienced by participants, should be studied both quantitatively and qualitatively in future intervention studies. Moreover, using the suggestions of Skaar et al. (2016) to place students into three tiers of PBIS with different goals based on students’ past experiences and needs may be useful to explore the program duration, type of instructor, and type of curriculums within each tier. Lastly, few forgiveness education intervention studies have attempted to measure contextual changes such as cooperation, academic outcomes, and harmony. It is possible that reduced anger and increased forgiveness may lead to changes at the school, community, and societal levels. Thus, contextual measures should be implemented in studies to further explore these potential changes.

## CONCLUSIONS

The present meta‐analytic review demonstrates that forgiveness education interventions effectively decrease anger and increase forgiveness among children and adolescents when compared with those who did not receive forgiveness education interventions. In addition, results indicated that forgiveness education interventions have robust effects that remain even after the termination of the program. Although more studies are needed to produce more generalizable results, it appears that forgiveness education interventions are effective regardless of whether participants have experienced severe or mild offenses or attend schools in economically disadvantaged areas. Overall, results lend support to the idea that children and adolescents experience hurt and conflicts in interpersonal relationships and may benefit from learning more about what forgiveness is and the process of how to forgive.

## CONFLICT OF INTEREST

We have no conflicts of interest to disclose.

## Supporting information

Appendix
