# Supplementary material for: A meta‐analysis of forgiveness education interventions’ effects on forgiveness and anger in children and adolescents
Source: Child Dev. 2022 Apr 22;93(5):1249–69. doi: 10.1111/cdev.13771 (PMC9544775; doi:10.1111/cdev.13771)
Supplement: Supplementary file 1 — Appendix [file CDEV-93-1249-s001.docx]

**Coding Manual & Other Appendices**

**Contents**

[**Code Reference for “Data_CD.xlsx”** 2](#_gjdgxs)

[**APPENDIX A**:](#_1fob9te) [**Coding Procedure for Forgiveness Education Meta-Analysis** 3](#_3znysh7)

[**APPENDIX B**:](#_2et92p0) [**Forgiveness Education Meta Coding Sheet** 8](#_tyjcwt)

[**APPENDIX C: Outcome Measure Descriptions & References**  12](#_3dy6vkm)

**APPENDIX D: Results with the inclusion of Gambaro et al., (2008)** 14

# **Code Reference for “Data_for_journal.xlsx”**

Access Data here: <https://osf.io/94kez/?view_only=957d99b69a8b4be29fc912f8a8d54809>

**Variables for “Moderators” spreadsheet**

| **ID** | Article author(s), article publication year |
| --- | --- |
| **G** | Effect size |
| **VAR** | Effect size variance |
| **modVAR** | Modified effect size variance |
| **Long_Cite** | Full article citation |
| **Study_ID** | Assigned number per study |
| **Pub_Status** | Publication Status |
| **Comparison_group_type** | Type of comparison group |
| **female** | Percentage of female participants |
| **mean_age** | Average age of all participants |
| **mean_grade** | Average grade level of all participants |
| **Grade_recode** | Grade thresholds by academic levels (1^st^ through 3^rd^ etc.) |
| **Income_Level** | income level of area in which the school is located |
| **severity** | Severity of the offense that participant experienced |
| **Instructor_type** | Facilitator for the forgiveness education program |
| **sessions** | Number of sessions in the program |
| **Session_length** | Session length in minutes |
| **total_time** | Sessions * session length in minutes |
| **total_hours** | Sessions * session length in hours |
| **sessions_per_week** | Sessions per week |
| **number_of_weeks** | Number of weeks program ran for |
| **Typical** | Typical student population vs. special student population |
| **Story** | Type of curriculum |

# **APPENDIX A**

# **Coding Procedure for Forgiveness Education Meta-Analysis**

Coding of studies for this meta-analysis (M-A) will proceed in three phases.  First, coders should determine whether the study in question meets the inclusion criteria for the M-A. If it does, coders next read the method section carefully to determine the appropriate codes for the study on a number of features of interest for the M-A.  Finally, after coding for study characteristics is complete, we will return to each study focusing on the Results section, to code one or more effect sizes representing treatment outcome(s) for that study.

**Phase 1:  Does the study meet inclusion criteria?**

- We are looking for studies about forgiveness psychoeducation not forgiveness psychotherapy.
- Education must be taught by a teacher or instructor or someone meant to take the role of a teacher. This could even be a psychologist as long as they are teaching and not implementing a clinical therapy.
- We are only looking at child through adolescent population. The majority of the population should be between the ages of 4-19 or kindergarten through high school. During the period when a child is in school, is when educational curriculums are most relevant.
- Set curriculum for the psychoeducation. This mean that there is planning and thought that went into the curriculum. Not just trial and error, a book group, or an observation.
- Group treatment rather than individual to be similar to what would occur in classroom.

**Forgiveness Education Meta-Analysis**

**Category Codes and Descriptions**

**I. Characteristics of Studies**

1. **Publication status**
2. Published journal article
3. Unpublished article (non-dissertation)
4. Dissertation (can be published or unpublished)

1. **Number of groups (1, 2, or 3+)**
2. Single group (both pre- and post-scores assessed)
3. Two groups (type of comparison coded in category C below)
4. More than two groups

**Comment:**  This information should be in the Procedures subsection of Method.  If not, check whether there is an Analysis subsection of Method.  If not, you may need to look in Results.

1. **Type of comparison group**

0 = None (single group study)

1. No-treatment control (e.g., wait-list control group)
2. Placebo control (minimal intervention to control for non-specific factors)
3. Active treatment condition
4. More than one type of comparison group (e.g., placebo as well as wait-list)

**Comment:**This information should also be found in Procedures, where the nature of the groups will be specified.  Placebo treatments should be “non-specific,” in the sense that they are not forgiveness-focused psychoeducation. They should also not be bona fide psychoeducations like the active treatment. So, if the comparison group gets a validated psychoeducation (which does not focus on forgiveness), we would consider this a treatment condition (code = 3) rather than a placebo control (code = 2).  If code=4 and there are more than one groups then write down what each group type is (placebo, control, etc.)

**Experimental Method of assignment (random or nonrandom)**

           Write “N/A” for not applicable-- there is no comparison group

1=Participants randomized (by classroom or group)

2=Participants not randomized (by classroom or group )

3=Unspecified

**Comment:**  This information may not be clearly stated for some studies, so some reading between the lines may be necessary.  Some guidelines for each category code:

Code 1:  Only assign a code of “1” if the Method section contains an explicit reference to “random” assignment by classroom or otherwise.  (Usually this information should be in the Procedures subsection.)  Unless random assignment is specified, we’ll assume the study is nonexperimental.

Code 2:Participants are assigned to the conditions by the researcher, but random assignment is not specified.  For example, participants may be assigned to treatments consecutively based on the order in which they registered for the study. Studies should receive a code of “2” if participants have a choice about their treatment condition “2” is also appropriate

- 1. **Characteristics of Sample**

1. **Number of participants**

(Enter total *N* for all study participants who **received the treatment/psychoeducation**)

**8a. N female**

[Enter the number of all participants who were female in the TX group]

**Comment:**  Information should be in Participants subsection of Method.  This is the information most likely to be available from all studies.

1. **Mean age**

[Enter average age of study participants.]

**Comment:**  Some studies will not report the mean age, but they will report other information that gives some idea of what the typical age of participants was.  For example, for a study that does not report the mean age but does report the median age, **enter the median age.**  For a study that does not report the mean age but does report the age range, **enter the midpoint between the minimum and maximum ages.**  If too little information on age is provided even to make a crude estimate of the mean, code this as NA, and note any relevant study features (e.g., participants are school-age children, so mean age must be < 18) in the margin or comments section of the coding sheet. If the grade range, mean, median, or mode is reported, include this as well.

1. **Mean grade**

[Enter average grade of study participants.]

**Comment:**  If a grade is from another country, such as Ireland, it will need to be translate into the US system of grades. For example, year 2 or primary 2 in Ireland equates to first grade in the US.

**Also, by academic level**

1= lower primary (1-3)

2= upper primary (4-5)

3= middle school (6-8)

4= high school (9-12)

**12. Income Level**

1=disadvantaged

2=nondisadvantaged

3=unspecified

**Comment:**Information should be in Participants subsection of Method. For code 1 “disadvantaged” first look to see if the article mentioned the school was in a low-income or troubled area. If you have the location of the school, pleas look up to see it the school is in a town that is below the national median income. You can do this through census data. If the town of the school or description of the economic status of the area is not mentioned, please write in a code 3 so that we can email the study’s author.

1. **Severity of offense:**

 0=Not stated

1=Mild experience

2= Severe experience

**Comment:**  Assume mild is “easy to regain what is lost”(i.e. verbal insult and light injury). Assume a sever experience is “irreversible or difficult to regain” (i.e. loss of body part, loss of a family member, abuse or victimization)

1. **Special Population**

1= Normal/typical

2= Special Population

Comment: Participants are "typical" school students without a determined commonality (other than age or grade, gender) Example: If students were randomly selected form a local school in their given country, it could be this group of students. Participants are "special" students, if they were selected because they possess a determined commonality (other than age, grade, gender) Example: If students were randomly selected form a local school in their given country, it could not be this group of students (share the commonality of mental health diagnosis, a trauma, a shared situation like being a child of divorce or bullied)

**III. Treatment Characteristics**

1. **Instructor Type**

1=Instructor is a teacher or school counselor

2=Instructor was a psychologist

**Comment:**Psychologist does not have to be a licensed clinician; this could also refer to a PhD or Masters in psychology. Instructor refers to the educator, most likely a school teacher.

1. **Curriculum**

1= Enright’s model using story-based curricula

2= Enright’s model using process-based curricula

3= REACH model

**Comment:**Enright’s process model refers to the 4-phase process model of forgiveness (with 20 steps). REACH model refers to Worthington’s REACH forgiveness model (5 steps). Enright’s story-based curriculum on International Forgiveness website. Enright.” This information should also be found in Procedures subsection of the method. Characteristic of Enright’s story-based model is the idea that forgiveness is distinguished from reconciliation and that primary goal of the psychoeducation is learning about forgiveness rather than “forgiving.”

1. **Number of sessions (treatment as designed)**

[Enter number of sessions offered for treatment group(s).  If there is more than one active treatment group, it is usually possible to identify one as the focal treatment (e.g., and experimental treatment that is being compared to an existing or usual method of treatment).  In this case, the entry should reflect the number of sessions for the focal treatment group.]

1. **Session length (minutes)**

[Enter the length of each session (or the average length, in the unusual event that the

sessions are different lengths)

1. **Number of weeks**

[Enter number of weeks of treatment for treatment group(s).]

**Comment:** if the study includes a mention of weeks actually attended vs. intended for participant to attend, make a note.

# **APPENDIX B**

# **Forgiveness Education Meta Coding Sheet**

*Inclusion Criteria:*

Forgiveness psychoeducation v. psychotherapy---

Taught by a teacher or instructor/someone meant to take the role of a teacher:

Yes(1) No (0)

Child- Adolescent population (majority ages 4-19):

Yes(1) No(0)

Set curriculum for the psychoeducation

Yes(1) No(0)

Group treatment

Yes(1) No(0)

*If any no, discontinue coding*

1. ***Study Characteristics***

| **1.** | **Article Name (1^st^ six words)** |  |
| --- | --- | --- |
| **2.** | **Article Authors (1^st^ and 2^nd^ authors)** |  |
| **3.** | **Article year** |  |
| **4.** | **Pub status** | 1 2 3  Journal article Unpublished Dissertation |
| **5.** | **# of groups** | 1 2 3  single two 3+ |
| **6.** | **Comparison**  **group type** | 0 1 2 3 4  None No-Tx. control Placebo Active Tx >1 Type |
| **7.** | **Experimental Method** | Randomized (by classroom/group)=1  Not randomized (by classroom/group)= 2 unspecified=3 |

***II. Sample Characteristics***

| **8.** | **Sample Size**: N (total in treatment group) = | n(female)= | n(male)= | | | | | | |
| --- | --- | --- | --- | --- | --- | --- | --- | --- | --- |
| **9**. | Mean Age of Children= | Age range of children= | | | | | | | |
| **10.** | **Mean Grade:** | | | | | | | | |
|  | **Ethnicity:** Describe race percentages, description of country and ethnicity, if minority is oppressed | | | | | | |  |  |
| **12.** | **Income Level** | 1= “predominantly low-income”/disadvantaged (below national median)  2= not disadvantaged (at national median or above) | | | | |  | | |
|  | Describe Percentages included in the report | | | | | | |  |  |
|  | Describe type of offense |  | | |  | | | | |
| **13.** | **School Type**: 1= private school 2=public school 3=not a school | | |  | |  | | | |
| **14.** | **Severity of the Offense**: 0=Not stated 1=Mild experience 2=Severe experience | | |  | |  | | | |

***III. Treatment Characteristics***

|  | Name of TX/  Author(s) and Year |  |
| --- | --- | --- |
| **15.** | **Instructor Type**  Teacher/school counselor = 1 Psychologist=2 |  |
| **16.** | **Distinction in curriculum goals*:***  Enright’s model using story curricula =1  Enright’s model using process curricula =2  Worthington’s model using REACH curricula =3  Other = 4 |  |
| **17.** | **Number of sessions:** |  |
|  | **Session Length:** |  |
|  | **Number weeks:** |  |
| **18.** | Special Population  1=no  2=yes |  |

**Effect Size Coding Sheet**

**Effect Size #1—Characteristics (circle one for each category):**

|  | (a) | (b) | (c) | (d) | (e) | (f) | (g) | (f) |
| --- | --- | --- | --- | --- | --- | --- | --- | --- |
| Design | Tx-NoTx | Tx-alternative | Tx only |  |  |  |  |  |
| Timing | pre-post | pre-FU |  |  |  |  |  |  |
| DV type (Circle) | Forgiveness | Anger | Depression | Self-esteem | School performance: Grade | School Performance: Discipline | Hope | Other |

**Effect Size #1--Data**

| Group | **n/grp** | **Mpre** | **SDpre** | **Mpost** | **SDpost** | (t-post) | (F-post) |
| --- | --- | --- | --- | --- | --- | --- | --- |
| Tx |  |  |  |  |  |  |  |
| Comp |  |  |  |  |  | *df:* | *df:* |
|  |  |  |  |  |  | *p:* | *p:* |

Comments:

| Name of Measure /  Author(s) and Year |  |
| --- | --- |
| Reporter | 1=Self 2=Other (CIRCLE: Parent Teacher Observer Interviewer Other) |
| Modification Status | 1=established and non-modified, 2=established and modified, 3=novel or unestablished |
| Type of Scale | 1=Objective Rating Scale (e.g., Questionnaire with quantitative options)  2=Subjective Rating (e.g., Observation, open-ended questions that are later coded) 3=Other |

|  | (a) | (b) | (c) | (d) | (e) | (f) | (g) | (f) |
| --- | --- | --- | --- | --- | --- | --- | --- | --- |
| Design | Tx-NoTx | Tx-alternative | Tx only |  |  |  |  |  |
| Timing | pre-post | pre-FU |  |  |  |  |  |  |
| DV type | Forgiveness | Anger | Depression | Self-esteem | School performance: Grade | School Performance: Discipline | Hope | Other |

**Effect Size #2—Characteristics (circle one for each category):**

**Effect Size #2--Data**

| Group | **n/grp** | **Mpre** | **SDpre** | **Mpost** | **SDpost** | (t-post) | (F-post) |
| --- | --- | --- | --- | --- | --- | --- | --- |
| Tx |  |  |  |  |  |  |  |
| Comp |  |  |  |  |  | *df:* | *df:* |
|  |  |  |  |  |  | *p:* | *p:* |

# **APPENDIX C**

**Outcome Measure Descriptions & References**

**Forgiveness Measures**

Of the initial twenty studies, fifteen studies (*k* = 15) measured forgiveness outcomes. Most of the studies (*k* = 14, or ~93%) used the Enright Forgiveness Inventory (EFI) or the EFI-Child version. The EFI (Subkoviak et al., 1995) and EFI-C (Enright & Neto, 2004) measure positive and negative affect, behaviors, and cognitions towards a person who offends. LaTurner (2006) administered the Heartland Forgiveness Scale and a self-developed scale. The Heartland Forgiveness scale measures a person’s dispositional forgiveness, or the general tendency to be forgiving, rather than forgiveness of a particular event or person (Thompson et al., 2005). LaTurner’s self-developed “situational forgiveness scale” also relates to dispositional forgiveness. The scale presents the participant with hypothetical situations and asks the participant to rate their likeliness of forgiving. One study (Hui & Chau, 2009) used the EFI and a one-item forgiveness scale that asked if the participant had forgiven the person who offends. All outcome measures were self-report measures.

**Anger Measures**

Thirteen studies (*k* = 13) measured anger outcomes. A total of 8 different self-report scales were used. The most prevalent scale, used by 6 studies (~ 46%), was the Beck Anger Inventory for Youth (Beck et al., 2001). The scale assesses perceptions of mistreatment, negative thoughts and feelings about others and physiological arousal. The Sate-Trait anger scale (STAXI-2; Spielberger et al., 1983) was used by two studies (Park et al., 2013; Bonab et al., 2020). Bonab (2021) utilized the state anger (individual’s current condition), trait anger (anger experienced over time), and anger expression (how often angry feeling are expressed verbally of physically) subscales. Rahman et al. (2018) indigenously developed a scale similar to the STAXI-2, that contained subscales for state and expressive anger. Park et al. (2013) only utilized the expression subscale. Shecthman et al. (2009) used the Transgression-Related Motivations Inventory (TRIM; McCullough et al., 1998) in order to measure motivation to seek revenge or to avoid a particular person. Similarly, LaTurner (2006) used the Vengeance Scale (Stuckless & Goranson, 1992) to measure a person’s attitude toward revenge. Beck (2005) used the The Aggression Questionnaire (Buss & Perry, 1992) to assess anger and aggression in individuals. Lastly, Vassilopoulos et al. (2020) used the Anger Expression Scale for Children – Trait (AESC-T;Steele, Legerski, Nelson, & Phipps, 2007). AESC-T trait anger subscale is a 10-item questionnaire that measures anger expression and hostility in children aged 6–18 years.

**References**

Beck, J. S., Beck, A. T., & Jolly, J. B. (2001). *BECK youth inventories of emotional & social impairment: Depression inventory for youth, anxiety inventory for youth, anger inventory for youth, disruptive behavior inventory for youth, self-concept inventory for youth: Manual*. Psychological Corporation.

Buss, A H., & Perry, M. (1992). The Aggression Questionnaire. *Journal of Personality and*

*Social Psychology*, *64*, 452-459.

Enright, R. D. &  Neto, J. R. (2004). *Enright Forgiveness Inventory for Children*. International Forgiveness Institute Inc. <https://internationalforgiveness.com/product/the-enright-forgiveness-inventory-for-children/>

McCullough, M. E., Rachal, K. C., Sandage, S. J., Worthington Jr, E. L., Brown, S. W., & Hight, T. L. (1998). Interpersonal forgiving in close relationships: II. Theoretical elaboration and measurement. *Journal of personality and social psychology*, *75*, 1586.

Spielberger, C. D., Jacobs, G., Russell, S., & Crane, R. S. (1983). Assessment of anger: The state-trait anger scale. *Advances in personality assessment*, *2*, 161-189.

Steele, R. G., Legerski, J.-P., Nelson, T. D., & Phipps, S. (2007). The anger expression scale for children: Initial validation among healthy children and children with cancer. *Journal of Pediatric Psychology*, *34*, 51–62.

Stuckless, N., & Goranson, R. (1992). The vengeance scale: Development of a measure of attitudes toward revenge. *Journal of social behavior and personality*, *7*, 25.

Subkoviak, M. J., Enright, R. D., Wu, C. R., Gassin, E. A., Freedman, S., Olson, L. M., & Sarinopoulos, I. (1995). Measuring interpersonal forgiveness in late adolescence and middle adulthood. *Journal of adolescence*, *18*, 641-655.

Thompson, L. Y., Snyder, C. R., Hoffman, L., Michael, S. T., Rasmussen, H. N., Billings, L. S., ... & Roberts, D. E. (2005). Dispositional forgiveness of self, others, and situations. *Journal of personality*, *73*, 313-360.

**APPENDIX D**

**Results with Gambaro et al. (2008).**


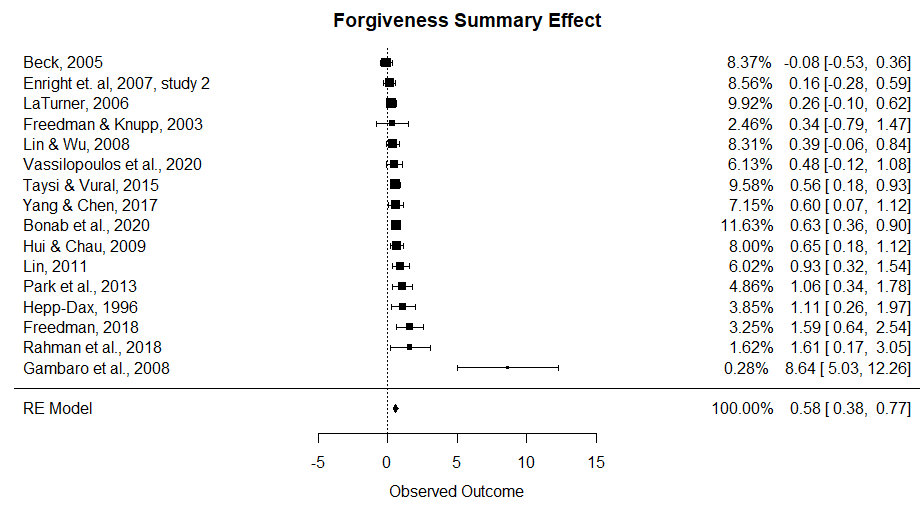


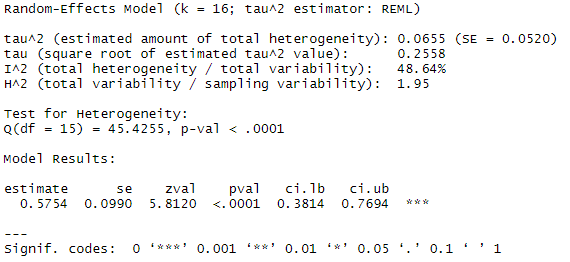


Anger and forgiveness Combined (including Gambaro, 2008):


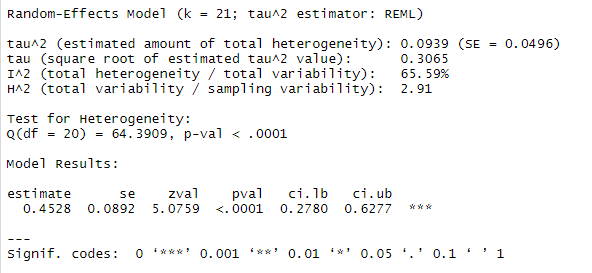


**Publication Bias**

All effect sizes of pooled point estimates (of forgiveness and anger measures) are displayed in a standard funnel plot in 4a. To assess the publication bias, we first ran the extension of Egger’s regression test (Egger et al., 1997; Rothstein et al., 2005) by calculating the weighted regression of the effect sizes on their standard errors, weighted by the inverse of their variances. The weighted regression slope is *β*_1_ = 0.03 (95% CI [-0.36, 0.43]), which indicates the skewness of the funnel plot is possibly due to the publication bias (Lin & Chu, 2018). Second, we conducted sensitivity analysis to check the severity of publication bias, which is the ratio of how much more likely are “statistically significant” results to be published than negative or “nonsignificant” results (Mathur & VanderWeele, 2020). Figure 4a shows right-skewed independent effect sizes generated with publication bias. The significance funnel plot in Figure 4b suggests a positive correlation between the effect sizes and their standard errors. This approach assumes that such correlation arises from selection is due to publication bias rather than to correlation between the effect sizes and standard errors in the underlying population (Mathur & VanderWeele, 2020). The worst-case estimate (represented by the grey dot in Figure 4b) from the meta-analyses of 8 non-affirmative pooled point estimates is *g* = 0.07 (95% CI [-0.08, 0.21]). To estimate the severity of publication bias, we attenuated the pooled point estimates to the null and to a non-null effect size of 0.1 (Mathur & VanderWeele, 2020). Under the random-effect specification, for the publication bias to attenuate the point estimate to the null, it is impossible for the publication bias to attenuate the pooled point estimate to the null. For the publication bias to attenuate the pooled point estimate to 0.1, affirmative studies would need to be at least 32-fold more likely to be published than non-affirmative studies. Thus, the overall conclusion is that, regardless of the severity of publication bias, this meta-analysis provides strong evidence for an average effect in the observed direction, albeit possibly of small size (Mathur & VanderWeele, 2020).


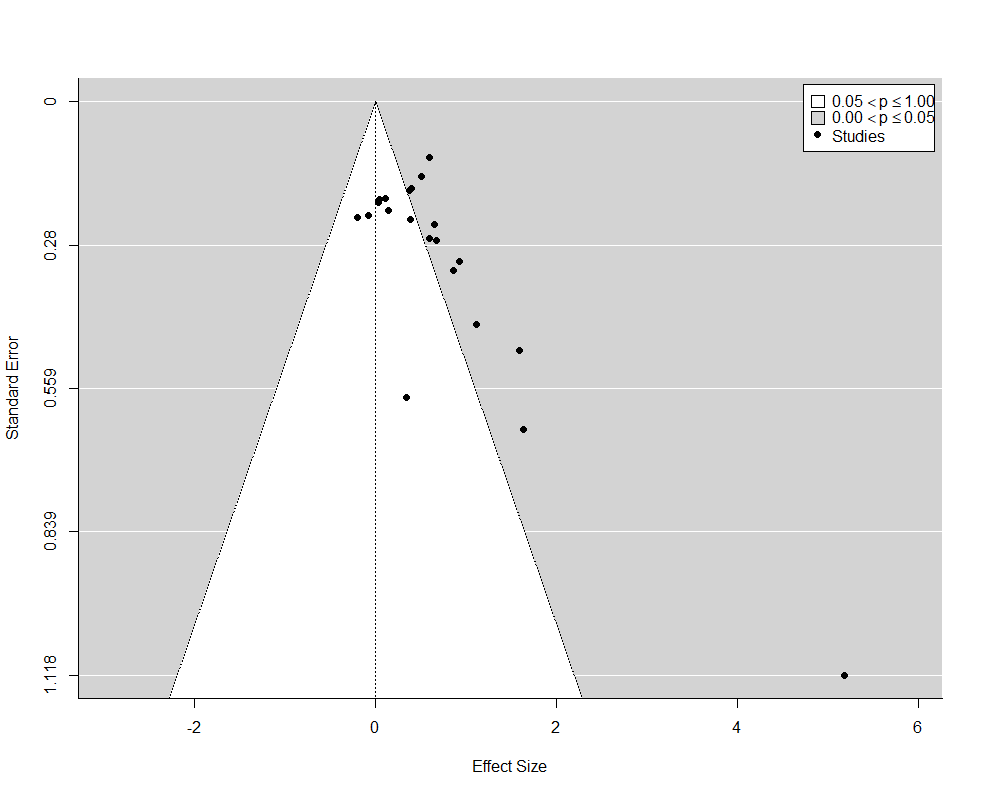

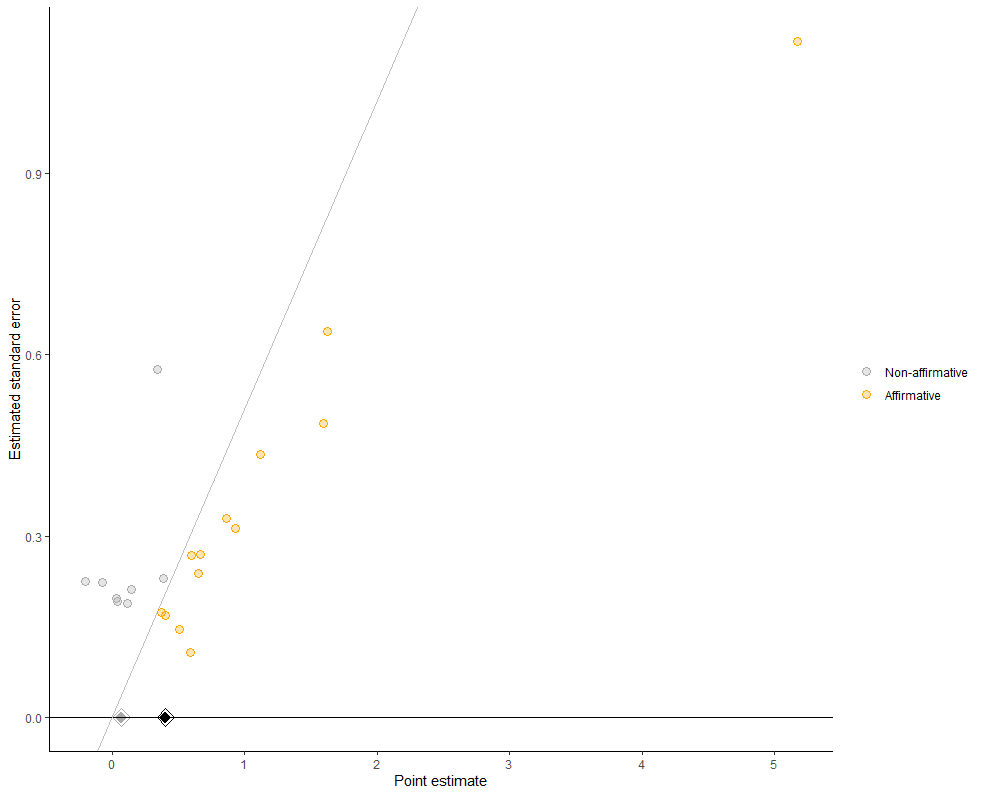


                                    (a)                                                   (b)

*Figure 4*. (a) Standard funnel plot *versus* (b) significance funnel plot for data generated with publication bias and with right-skewed population effect sizes. Effect sizes lying on the diagonal line have exactly *p* = 0.05. Grey dot: non-affirmative; orange dot: affirmative; black diamond: pooled point estimates within all studies; gray diamond: pooled point estimates within only the studies with non-affirmative results.
